# Body length changes for Atlantic salmon (*Salmo salar*) over five decades exhibit weak spatial synchrony over a broad latitudinal gradient

**DOI:** 10.1002/ece3.11538

**Published:** 2024-06-09

**Authors:** Tara L. Imlay, Cindy Breau, Guillaume J. R. Dauphin, Gérald Chaput, Julien April, Scott Douglas, J. Derek Hogan, Sherise McWilliam, Daniela Notte, Martha J. Robertson, Andrew Taylor, Kari Underhill, Laura K. Weir

**Affiliations:** ^1^ Fisheries and Oceans Canada Moncton New Brunswick Canada; ^2^ Ministère de l'Environnement, de la Lutte contre les changements climatiques, de la Faune et des Parcs Québec Québec Canada; ^3^ Fisheries and Oceans Canada French Village New Brunswick Canada; ^4^ Fisheries and Oceans Canada Dartmouth Nova Scotia Canada; ^5^ Fisheries and Oceans Canada St. John's Newfoundland and Labrador Canada; ^6^ Department of Biology Saint Mary's University Halifax Nova Scotia Canada

**Keywords:** climate, dynamic factor analysis, fisheries, fork length, habitat availability, size‐at‐age, spatial concordance, thermal habitat

## Abstract

Understanding the factors that drive spatial synchrony among populations or species is important for management and recovery of populations. The range‐wide declines in Atlantic salmon (*Salmo salar*) populations may be the result of broad‐scale changes in the marine environment. Salmon undergo rapid growth in the ocean; therefore changing marine conditions may affect body size and fecundity estimates used to evaluate whether stock reference points are met. Using a dataset that spanned five decades, 172,268 individuals, and 19 rivers throughout Eastern Canada, we investigated the occurrence of spatial synchrony in changes in the body size of returning wild adult Atlantic salmon. Body size was then related to conditions in the marine environment (i.e., climate indices, thermal habitat availability, food availability, density‐dependence, and fisheries exploitation rates) that may act on all populations during the ocean feeding phase of their life cycle. Body size increased during the 1980s and 1990s for salmon that returned to rivers after one (1SW) or two winters at sea (2SW); however, significant changes were only observed for 1SW and/or 2SW in some mid‐latitude and northern rivers (10/13 rivers with 10 of more years of data during these decades) and not in southern rivers (0/2), suggesting weak spatial synchrony across Eastern Canada. For 1SW salmon in nine rivers, body size was longer when fisheries exploitation rates were lower. For 2SW salmon, body size was longer when suitable thermal habitat was more abundant (significant for 3/8 rivers) and the Atlantic Multidecadal Oscillation was higher (i.e., warmer sea surface temperatures; significant for 4/8 rivers). Overall, the weak spatial synchrony and variable effects of covariates on body size across rivers suggest that changes in Atlantic salmon body size may not be solely driven by shared conditions in the marine environment. Regardless, body size changes may have consequences for population management and recovery through the relationship between size and fecundity.

## Graphical abstract

The fork length of Atlantic salmon (*Salmo salar*) increased during the 1980s and 1990s for six of 19 rivers in Eastern Canada. Fork length changes were related to exploitation rates from size‐selective fisheries and temperature for salmon that spent 1 or 2 years in the marine environment, respectively.

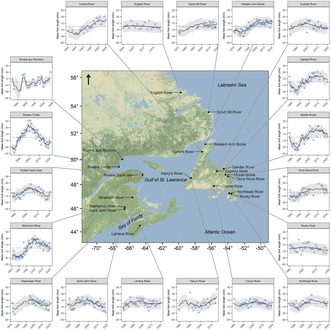

## 1 INTRODUCTION

Spatial synchrony is a common pattern of change through time among geographically distinct populations or species. Spatial synchrony can arise as the result of gene flow, trophic interactions, and/or shared environmental conditions (Bjørnstad et al., 1999; Moran, 1953; Walter et al., 2017). In the latter case, conditions that drive synchrony can occur over broad spatial scales and simultaneously affect multiple populations to a greater degree than localized conditions; alternatively, shared conditions can affect populations when they are temporarily concentrated in more discrete locations (Lloyd & May, 1999). When spatial synchrony is observed, it can provide insight into common or shared driver(s) of population change and enhance the efficacy of efforts to manage and conserve species and ecosystems across broad spatial scales. Further, when spatial synchrony occurs, it can provide insight on the relevant scale at which populations should be managed. Additionally, as indicated by the Portfolio effect (Figge, 2004; Schindler et al., 2015), high levels of synchrony may indicate relatively lower levels of life‐history diversity across populations compared to populations that vary asynchronously. Synchronous population dynamics can signal a decrease in the resilience of populations to environmental change (Hilborn et al., 2003; Moore et al., 2014; Schindler et al., 2010). Recent studies have documented synchronized patterns of change in range shifts (e.g., Santos et al., 2015), the timing of life cycle stages (e.g., Iler et al., 2021) and body size changes (e.g., Büntgen et al., 2014); below we focus on body size changes as this is an important metric for the management of many species because of its relationship with fitness.

Broad‐scale changes in body size can occur due to different abiotic and biotic factors. For example, warming global temperatures are expected to increase metabolic rates for ectotherms, increasing growth rates, and lead to shared patterns of reduced body size and maturity at younger ages (Daufresne et al., 2009; Gardner et al., 2011; Sheridan & Bickford, 2011). Indeed, broad‐scale declines in body size linked to warming temperatures have been found in several taxa, including insects, amphibians, and fish (Blanckenhorn, 2015; Caruso et al., 2014; Daufresne et al., 2009). Similarly, changes in suitable habitat availability (i.e., reductions in the available area or volume and/or increased fragmentation) have resulted in changes in body size to optimize survival and/or reproductive output (Letcher et al., 2007; Schmidt & Jensen, 2003; Warzecha et al., 2016). Trophic interactions can act as a synchronizing factor (Liebhold et al., 2004), with reductions in growth or body size during periods of low food availability (Anderson & Sabado, 1995; Büntgen et al., 2014; Yom‐Tov et al., 2007). In addition, density‐dependent relationships can result in lower growth rates and smaller body sizes when populations are large (Andersen et al., 2017; Bonenfant et al., 2009; Lorenzen & Enberg, 2002), and these density‐dependent processes may also be a synchronizing factor when they are shared across populations (Hugueny, 2006; Liebhold et al., 2004; Walter et al., 2017). Finally, size‐ or age‐selective mortality from hunting or fishing (i.e., harvest of the largest or oldest individuals) may result in populations of smaller and/or younger individuals (Allendorf & Hard, 2009; Fenberg & Roy, 2008; Law, 2000; ter Hofstede & Rijnsdorp, 2011). The relationships between the factors that drive body size changes are often complex, with more than one factor involved in observed changes in body size across populations (Oke et al., 2020). The consequences of body size changes for many populations are not well understood, but for exploited species where body size is correlated with fecundity and yield, reduced body size can have consequences for the sustainable management and recovery of depleted populations (Allendorf & Hard, 2009; Audzijonyte et al., 2013). Thus, identifying the factors that drive body size changes is key to managing species and populations.

Previous studies on adult salmonids have investigated body size changes across populations and the drivers of these changes; however, in most studies, synchrony across populations was not explicitly modeled. Changes in the adult body size of several species of Pacific salmon (*Oncorhynchus* spp.) have been documented across broad geographic areas (Jeffrey et al., 2017; Lewis et al., 2015; Ohlberger et al., 2023; Oke et al., 2020; Ricker, 1981). These reductions in body size have been linked to external factors such as changes in climate, inter‐ and intra‐specific density dependence, and size‐selective harvest (Jeffrey et al., 2017; Ohlberger et al., 2023; Oke et al., 2020; Ricker, 1981), as well as earlier maturity at a smaller size (Lewis et al., 2015; Oke et al., 2020, but see Ohlberger et al., 2023). For Atlantic salmon (*Salmo salar*), most prior studies have documented body size changes at a local (i.e., one or two rivers) or regional scale. For example, in two North American rivers, reduced body size was attributed to size‐selective salmon fisheries (Bielak & Power, 1986; Moore et al., 1995). More recently, some European Atlantic salmon populations have displayed slower growth rates (Long et al., 2023; Todd et al., 2021; Tréhin et al., 2021) and individuals that spent one winter at sea had smaller body size (Bacon et al., 2009; Jonsson et al., 2016; Todd et al., 2008). In the only study of multiple Atlantic salmon populations across a broad scale (i.e., 11° of latitude), Vollset et al. (2022) found a synchronous decrease in growth at sea in southern and mid‐latitude populations, but minimal changes in northern populations. These changes in growth rates and body size are linked to broad‐scale factors that also drive synchrony in other life‐history traits. Specifically, these studies suggest that the underlying mechanism is likely reduced food availability in the ocean that is correlated with increasing temperatures that subsequently affect body size (Long et al., 2023; Todd et al., 2021; Vollset et al., 2022).

Atlantic salmon populations have declined since the 1970s throughout the species' range in the North Atlantic Ocean (ICES, 2023). These declines are not well understood but may be the result of broad‐scale changes in the marine environment that have synchronous, range‐wide effects (Friedland et al., 2014; ICES, 2023, but see Pardo et al., 2021). Despite the substantial geographic variation in life‐history traits throughout their life cycle, previous studies have documented some level of spatial synchrony in Atlantic salmon population trends and demographic processes across broad geographic areas. This includes common long‐term trends in the abundance of adults (Friedland et al., 2014; Mills et al., 2013) and juveniles (Bouchard et al., 2022), productivity (Mills et al., 2013), survival during the early marine stage (i.e., post‐smolt stage) (Olmos et al., 2019, 2020, but see Pardo et al., 2021; Tirronen et al., 2022), the proportion of first‐time spawner age classes (Olmos et al., 2019) and repeat spawners compared to first‐time spawners (Bordeleau et al., 2019). Many of these synchronous processes have been linked to broad‐scale changes in the marine environment. For example, warmer temperatures in the North Atlantic Ocean are associated with an increase in phytoplankton and small zooplankton, declines in large copepods and euphausiids (important food sources for young salmon), and a decrease in abundance and productivity of Atlantic salmon (Beaugrand & Reid, 2003, 2012; Mills et al., 2013). Similarly, warmer temperatures and low food availability in feeding areas shared by multiple stocks are related to the reduced survival of young salmon during the early marine stage (Olmos et al., 2020).

In North America, spatial synchrony in body size changes for Atlantic salmon has not previously been investigated, nor have recent studies examined the relationships between body size changes and changing conditions in the marine environment. Therefore, we investigate spatial synchrony in body size changes of 19 Atlantic salmon populations throughout Eastern Canada over five decades. As these populations spanned most of the extant range of Atlantic salmon in North America, we considered whether changes in body size were synchronous throughout the range or if, similar to past studies on synchrony for other population traits (e.g., Bordeleau et al., 2019; Mills et al., 2013; Olmos et al., 2020), there was regional variation in body size changes. Also, we examined whether changes in body size were related to broad‐scale factors that could drive spatial synchrony. These factors were grouped into five broad categories: climate indices representing the physical oceanic environment, area of thermal habitat reflecting optimal metabolic rate and growth potential, indices of the prey base of salmon as a measure of food availability, total salmon population abundances to account for intra‐specific density dependence at sea, and size‐selective fisheries acting on mixed populations of salmon measured through exploitation rates. Identifying synchronous changes in body size has important implications for the management and recovery of salmon stocks in North America. This could include providing insight on the scale at which Eastern Canadian populations should be managed, as well as identifying management actions to address the factors driving body size changes. Further, stock reference points often include estimates of fecundity based on body size (ICES, 2023), and therefore, changes in the body size of salmon may affect whether stocks are able to meet reference points.

## 2 METHODS

### 2.1 Life history

Atlantic salmon is an obligate freshwater species with an optional anadromous life phase. After several years in freshwater post‐hatch, most young undergo smoltification, a process which includes physical, physiological, and behavioral changes that allow them to survive in the marine environment. At this time, young migrate from their natal rivers to common feeding areas in the North Atlantic Ocean as post‐smolts (Reddin, 2006). The largest and most rapid increases in body length and weight occur in the marine environment, particularly during the first summer at sea (O'Connell et al., 2006; Peyronnet et al., 2007; Tréhin et al., 2021). During the first winter at sea, salmon from Eastern Canada are generally distributed in the Labrador Sea and Grand Banks area (Dadswell et al., 2010; Reddin & Friedland, 1993). After one winter, a portion of the smolt cohort will mature and return to their natal rivers to spawn (straying rates vary but usually remain low; Jonsson et al., 2003; Keefer & Caudill, 2014); these individuals are referred to as one sea winter (1SW) first‐time spawners. The non‐maturing salmon migrate further to common feeding areas in the Labrador Sea, Greenland, and some travel to the Faroe Islands in the northeast Atlantic Ocean (Bradbury et al., 2021). Most survivors from this group mature and return to their natal rivers to spawn after the second winter at sea as two sea winter (2SW) first‐time spawners. The small remainder of each smolt cohort first spawns after only a few months at sea (Newfoundland), or three or more years at sea. In addition to the first‐time spawners, there is a small component (0%–24.7%; Bordeleau et al., 2019) of the population that is comprised of individuals that have spawned in two or more years, that is, repeat spawners. In our analyses below, we focus on body length changes for the two most common first‐time spawner age classes (1SW and 2SW). The ratio of these age classes varies among regions of Eastern Canada. Returning adults in rivers in Labrador, Quebec, New Brunswick, and Nova Scotia are a mix of 1SW and 2SW salmon, but in insular Newfoundland, salmon returns are primarily 1SW (Chaput et al., 2006; O'Connell et al., 2006).

### 2.2 Field data collection and compilation

**FIGURE 1:**
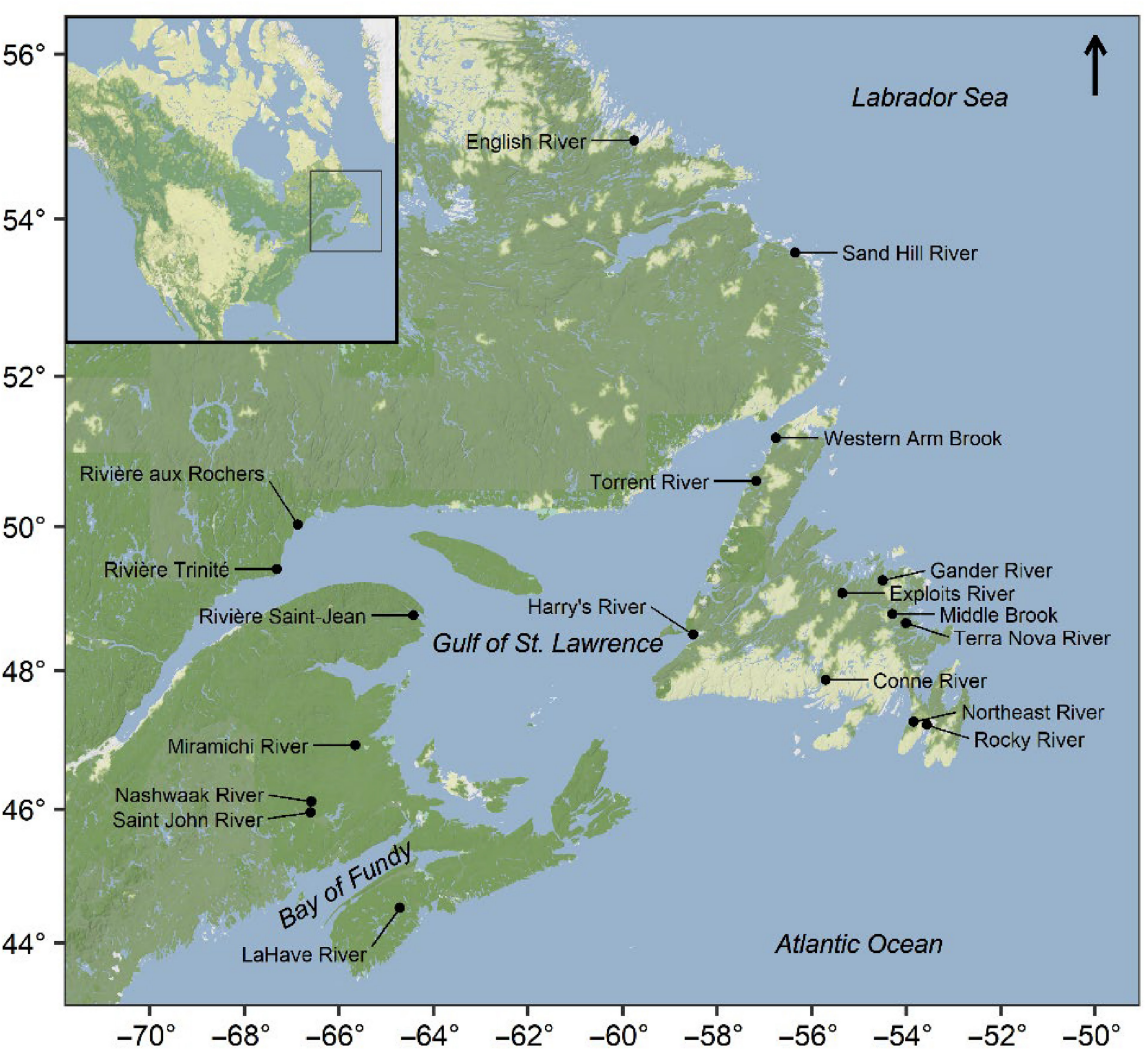
Locations of 19 Eastern Canadian rivers where Atlantic salmon were monitored between 1970 and 2022. The gray rectangle in the inset map outlines our study region in Eastern Canada. Base map tiles by Stamen Design, under CC BY 4.0. Data by OpenStreetMap, under ODbL.

**FIGURE 2:**
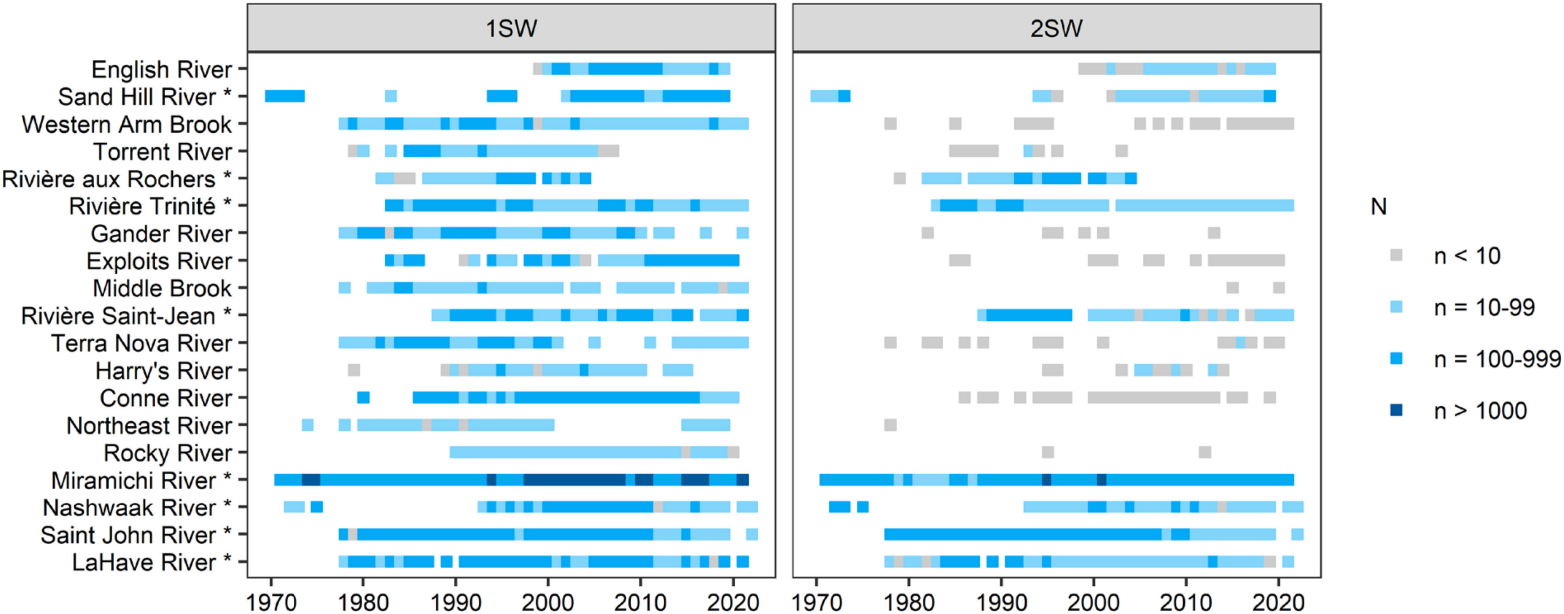
Data availability for 19 Eastern Canadian rivers where Atlantic salmon fork lengths were measured for at least 2 years between 1970 and 2022 after removing individuals from hatchery, smolt to adult supplementation, captive‐breeding, or aquaculture origin. Rivers are arranged by increasing latitude. Only rivers with an asterisk (*) were included in the 2SW dynamic factor analysis (DFA). Only data from 1971 (1SW) or 1972 (2SW) to 2021 were included in our analyses to match the temporal period of the covariates.

We compiled data on the fork lengths of 325,716 returning Atlantic salmon collected at 19 rivers (spanning over 10° of latitude) throughout Eastern Canada (Figure 1). These data were collected from 1970 to 2022, but the length and range of the time‐series were river‐specific (Figure 2). On average, there were 34.2 years of data for each river (range: 21–51 years). Field protocols and data collection methods varied by river (Table S1, Figure S1); only a small proportion of salmon included in our analysis were sampled with a size‐selective sampling method (i.e., gillnets: 0.06%–0.9%, Table S1). Salmon with markings that identified them as hatchery releases (11.9% of individuals), captive breeding or smolt‐to‐adult supplementation programs (0.006%), or known or suspected aquaculture (0.07%) were excluded from our analyses.

Our analysis focused on wild salmon from the two most common age classes: 1SW and 2SW first‐time spawners (64.7% and 26.5%, respectively, of 212,313 known age individuals). Aging adult salmon is done by reading scales which act as a recorder of somatic growth (Baglinière & Le Louarn, 1987; Panfili et al., 2002); experienced readers are able to identify slow and fast growth phases that are linked to winter and summer periods, respectively. Despite advances in the field (e.g., digital photography of scales and semi‐automated protocols), scale reading is still a challenging, labor‐ and time‐consuming task. As a result, only 62.6% of individuals in our dataset had a known age and fork length. In particular, we lacked data from scale reading for small salmon (<630 mm) in the Miramichi River from 2014 to 2018 and 2021; however, previous years of data indicate that this size class is predominantly 1SW salmon (mean across years: 99.5%, range 97.8%–100%). Therefore, in these years, we assumed that all individuals with a fork length less than 630 mm were 1SW, as there is minimal overlap in size around this threshold (Douglas et al., 2023). Additionally, for 1SW and 2SW age classes separately, we removed any years with fewer than 10 individuals per age class. Because 2SW salmon are infrequently observed in insular Newfoundland, no rivers from this area were included in the dataset for this age class. Finally, we included only rivers with 20 or more years of age and body size data from 1971 (1SW) or 1972 (2SW) to 2021; we removed the earliest and last years of data to match the period of data for our covariates.

Our final dataset was comprised of 123,907 1SW individuals from 19 rivers (mean ± SD: 32.7 ± 9.0 years/river, range 20–51 year/river) and 48,361 2SW individuals from eight rivers (33.4 ± 10.4 years/river, range 22–50 years/river) (Figure 2). Using these data, we calculated mean fork lengths for each river in each year for the dynamic factor analysis described below (see Section 2.4). We recognize that by using mean values, we ignore the uncertainty of the mean annual fork length values over the time‐series. We are also unable to address the within‐year variability in fork length, although the variation around mean fork length was generally consistent through time (Figures 5 and 6, Figure S2 and S3). Further, for many rivers, salmon had longer fork lengths later in the season (Figure S4); however, as sampling periods were similar and spanned the spawning run across most years for most rivers (Figure S1), trends should be due to changes in body size rather than changes in sampling periods. Additional summaries on variability in fork length across time and rivers are shown in Figures 5 and 6, Figures S2 and S3.

### 2.3 Covariates

**FIGURE 3:**
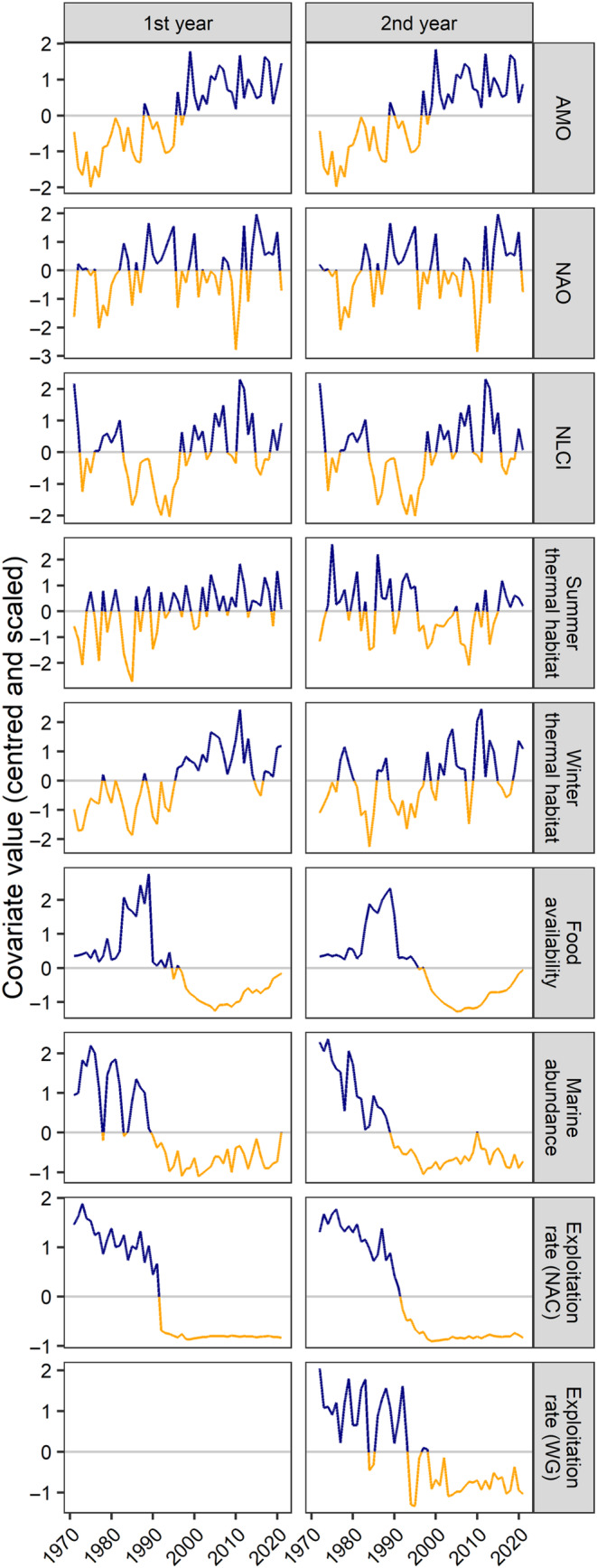
Normalized timeseries values for climate, including the Atlantic Multidecadal Oscillation (AMO), North Atlantic Oscillation (NAO), and Newfoundland and Labrador Climate Index (NLCI); summer and winter thermal habitat; food availability; pre‐fishery abundance as a measure of density dependence; and exploitation rates for fisheries in the North Atlantic Commission (NAC) and West Greenland (WG) during the first and second years at sea for Atlantic salmon. The values for food availability in the second year represent the combined value of the first and second years for 2SW salmon. Alternating orange and blue line colors indicate values below and above the mean for each covariate.

Although the growth and body size of salmon is likely due to a combination of factors experienced in freshwater and the ocean, we focused on broad‐scale covariates in the marine environment that were likely to affect all populations of Atlantic salmon in Eastern Canada in their shared marine feeding areas. As a result, we considered five groups of covariates that had the potential to result in synchronous changes in body size across populations: climate, thermal habitat, food availability, density‐dependence, and fisheries harvest (Table 1). Timeseries values for these covariates are in Figure 3 and the sources of covariate datasets are provided in Table S2.

**TABLE 1:** Covariates representing climate, thermal habitat, food availability, density‐dependence at sea, and fisheries exploitation rates that may affect the fork length of Atlantic salmon (*Salmo salar*) in Eastern Canada. For 2SW salmon, covariates included could affect growth during the first year at sea (most were the same as 1SW salmon) and those that could affect growth during the second year at sea.

Covariate group	Covariate	1SW	2SW	
		1st year	1st year	2nd year
Climate	Mean AMO	X	X	X
	Mean NAO (Dec–Mar)	X	X	X
	NLCI	X	X	X
Thermal habitat	Thermal habitat (Aug–Nov)	X	X	
	Thermal habitat (Dec–Apr)	X	X	X
	Thermal habitat (May–Nov)			X
Food availability	First principal component from PCA containing zooplankton and Capelin biomass	X	X	
Density‐dependence	Pre‐fishery abundance of 1SW cohort in NAC on 1 Jan	X	X	
	Pre‐fishery abundance of non‐maturing 1SW cohort in NAC & SNEAC on 1 Jan			X
Fisheries harvest	Exploitation rate of small salmon in NAC fisheries_year_	X		
	Exploitation rate of large salmon in NAC fisheries_year_			X
	Exploitation rate of NAC salmon in West Greenland fishery_year–1_			X

Abbreviations: AMO, Atlantic Multidecadal Oscillation; NAC, North American Commission; NAO, North Atlantic Oscillation; NLCI, Newfoundland and Labrador Climate Index; SNEAC, Southern Northeast Atlantic Commission.

#### 2.3.1 Climate

Climate indices represent physical components of the marine ecosystem and have been correlated with at‐sea growth for Atlantic salmon (Jensen et al., 2011; Jonsson & Jonsson, 2004b). However, the strength and direction of these relationships often vary across populations. To measure the effects of climate on Atlantic salmon body size, we retrieved monthly indices for the Atlantic Multidecadal Oscillation (AMO) and the North Atlantic Oscillation (NAO) indices. The AMO is a detrended index of sea surface temperature in the North Atlantic Ocean (from 0° to 70°) and represents long‐term variability in the ocean. During the early 1990s, AMO switched from a negative to a positive phase; the latter phase represents a period of warmer ocean temperatures (Figure 3). The NAO is measured as the atmospheric pressure difference between a low near Iceland and a high near the Azores and it fluctuates the most during the colder months of the year (Stenseth et al., 2003). Positive phases of NAO represent cooler temperatures and less precipitation in Canada, Greenland, and southern Europe, and warmer temperatures and more precipitation in the United States and northern Europe; the opposite patterns are observed during negative phases (Stenseth et al., 2003). For each year in our analysis, we calculated the mean AMO from January to December and the mean NAO from December to March (i.e., when NAO is most variable) with year corresponding to the January to March period. We also obtained annual values for the Newfoundland and Labrador Climate Index (NLCI). This index describes environmental conditions on the Newfoundland and Labrador shelf and the Northwest Atlantic Ocean by averaging anomalies for 10 metrics, including NAO (December to March), air and ocean temperatures at various depths, area of the cold intermediate layer (i.e., subsurface water <0°C), sea ice, icebergs, and salinity (Cyr & Galbraith, 2021). Positive values of the NLCI represent periods of warmer air and ocean temperatures, more precipitation, less sea ice, and fewer icebergs (Cyr & Galbraith, 2021).

#### 2.3.2 Thermal habitat

**FIGURE 4:**
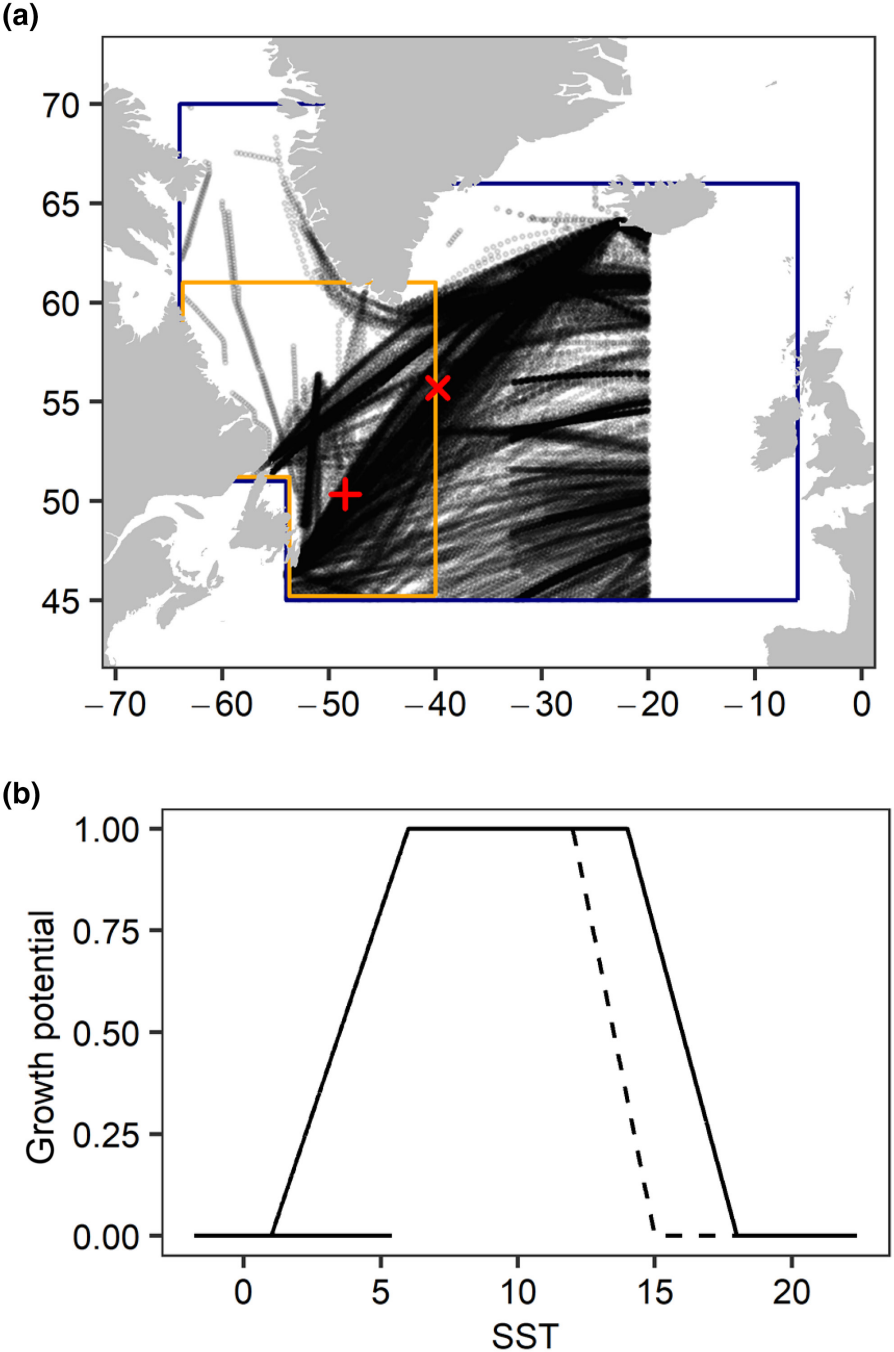
(a) Spatial regions used in the determination of thermal habitat and zooplankton for 1SW and 2SW Atlantic Salmon from Eastern Canada. The orange and blue polygons indicate the boundaries of the regions where salmon are found during their first and second years at sea, respectively; the full extent of the first‐year polygon to the south and west is displayed slightly inside of the second‐year polygon solely for visualization. The black circles indicate locations of zooplankton samples from the Continuous Plankton Recorder (CPR) survey from 1958 to 2021, and the red symbols indicate locations for estimated zooplankton indices in the first (+) and second (X) years. These were the most commonly sampled locations in the CPR dataset. (b) The growth potential curves for Atlantic salmon for all unique combinations of the sea ice concentration and sea surface temperature (SST) in the polygon for salmon during the second year at sea. The dashed line represents the difference in growth potential at different SSTs for salmon in their second year where it deviates from the growth potential for salmon in the first year. When sea ice concentrations were ≥0.5, 10 the growth potential of the habitat was 0, regardless of SST > 1°C.

For Atlantic salmon (and other ectotherms), the relationship between growth and temperature is not linear. Growth initially increases with warmer temperatures, but beyond an optimal temperature, growth rates decline (Handeland et al., 2008). At sea, salmon are likely to be found in waters of lower temperature than optimal for growth (Handeland et al., 2008), with warmer temperatures often associated with higher survival and growth (Friedland, 1998; Jensen et al., 2011; Olmos et al., 2020). However, other studies have also observed a negative effect of warmer temperatures on growth (Todd et al., 2008, 2021; Vollset et al., 2022). In our analysis, we choose to use a metric of thermal habitat that reflects the non‐linear relationships between growth and ocean temperature and therefore represents potential growth associated with ocean temperature. We retrieved the Hadley Centre Sea Ice and Sea Surface Temperature (HadISST1) dataset from the UK's Meteorological Office. HadISST1 uses reduced space optimal interpolation to reconstruct monthly values for sea ice concentration and sea surface temperature (SST) on a 1° × 1° spatial grid since 1871 (Rayner et al., 2003). We assumed salmon in their first year at sea were mostly found in the Atlantic Ocean and Labrador Sea in the area bounded by 45° to 61° latitude and −64° to −40° longitude (Figure 4a). To reflect the more northern distribution and trans‐oceanic movements of salmon during the second year at sea (Bradbury et al., 2021), we included this area and also extended the northern boundary to 70° in the Labrador Sea and 66° in the Irminger Sea and eastern Atlantic Ocean, and the eastern boundary to −6° W to capture the area around the Faroe Islands. Within each spatial region, we defined two temporal periods: summer and winter. The summer period for post‐smolts spanned August to November and during the second year, the summer period spanned May to November; the winter period spanned December to April in both years.

To calculate the thermal habitat metrics, we defined five sets of conditions for sea ice conditions and SST and used these to approximate the “curve” underlying the relationship between body size and temperature (Handeland et al., 2008). First, we defined the conditions where the growth potential was nil. For 1SW and 2SW, we assumed that SST <1°C (Vadboncoeur et al., 2023) and sea ice concentrations ≥0.5 represent conditions that were not suitable for growth and assigned degree blocks meeting these conditions a value of 0 (Figure 4b). Similarly, temperatures >18°C for individuals in the first year (Reddin et al., 2006) and >15°C for individuals in the second year (Strøm et al., 2020) were assigned a value of 0. Second, we defined the optimal temperatures for growth as 6–14°C for the first year (Crouse et al., 2022; Handeland et al., 2008; Vadboncoeur et al., 2023) and 6–12°C in the second year (Beauchamp, 2009); degree blocks with temperatures in the optimal ranges were assigned a value of 1. Third, between the temperature ranges above for either no or optional growth, we assumed a linear relationship between temperature and growth potential. Specifically, from 1 to 6°C, we assumed the value of the habitat would increase linearly from 0 to 1 (e.g., a degree block with an SST 2°C was assigned a value of 0.2), and from 14 to 18°C for the first year and 12–15°C for the second year, the value of the habitat would decrease linearly from 1 to 0. Lastly, we multiplied the weightings above by the area of the degree blocks (to correct for the curvature of the earth) and summed the resulting values within each spatial region and time‐period. This resulted in four metrics of thermal habitat (summer and winter in the 1st and 2nd years).

#### 2.3.3 Food availability

Salmon at sea are opportunistic foragers that feed on a variety of prey. Primary sources of food include zooplankton, like copepods, small crustaceans (e.g., euphausiids, hyperiid amphipods, and shrimp), and forage fish, like Capelin (*Mallotus villosus*) (Haugland et al., 2006; Jacobsen & Hansen, 2001). Diet is related to body size, with small 1SW salmon typically consuming smaller prey and larger 2SW salmon consuming larger prey (Jacobsen & Hansen, 2001). We developed annual indices of food abundance at two trophic levels, including: (1) zooplankton biomass consisting of copepods (e.g., *Calanus* sp.), euphausiids and hyperiid amphipods, with data from the continuous plankton recorder (CPR) survey (Richardson et al., 2006) (Figure 4a), to represent secondary productivity and 1SW diet (Haugland et al., 2006; Jacobsen & Hansen, 2001); and (2) Capelin biomass, to represent 1SW and 2SW diet (Jacobsen & Hansen, 2001). In the Supporting Information, we detail the steps to calculate annual biomass for zooplankton and Capelin.

To better represent the broad prey field consumed by Atlantic salmon in the ocean across different ages, we combined the annual values for zooplankton and Capelin biomass in a principal component analysis (PCA) to develop a single food availability index. For 1SW salmon, the index included zooplankton from the 1SW polygon and Capelin biomass in the subsequent year. For 2SW salmon, the same variables as the 1SW index during the first year at sea, as well as zooplankton from the 2SW in the second year and Capelin biomass in the subsequent year. As the first principal component explained 77.6% and 72.4% of the variability in zooplankton and capelin biomass for 1SW and 2SW salmon, we used these values as our final index of food availability for each age group. Negative values represent periods of low zooplankton and Capelin biomass (Table S4, Figure S5).

#### 2.3.4 Density‐dependence at sea

Several studies have reported density‐dependent effects on the marine growth of Pacific salmon (Jeffrey et al., 2017; Ohlberger et al., 2023; Oke et al., 2020) and other marine fish (Andersen et al., 2017; Lorenzen & Enberg, 2002; Rindorf et al., 2022; Zimmermann et al., 2018). Although at‐sea survival is considered density‐independent for Atlantic salmon (Jonsson & Jonsson, 2004b), Atlantic salmon in the Baltic Sea have been reported with larger body sizes in years with low abundance (Huusko & Hyvärinen, 2012). To our knowledge, density‐dependent relationships with body size have not been explicitly examined for Atlantic salmon in the North Atlantic Ocean. We developed a metric of abundance in the ocean using the pre‐fishery abundance (PFA) estimates for salmon at a time when they would be distributed on the feeding grounds in the Northwest Atlantic. The PFA values were obtained from the International Council for the Exploration of the Sea (ICES, 2023) compilations for the Canadian and United States of America stocks of the North American Commission (NAC) and for southern European stocks included in the Southern Northeast Atlantic Commission (SNEAC; France, southern and western Iceland, Ireland, and United Kingdom stocks). PFA for salmon from the NAC and SNEAC is reported as of 1 August and 1 January, respectively. Therefore, the PFA for North America was adjusted back to 1 January using an assumed mortality rate of 0.03 per month for 7 months (PFA*e^0.03*7^; ICES, 2002; Potter et al., 2003). PFA is estimated annually for two components within each smolt cohort: (1) the “maturing component” that includes salmon that mature and return to rivers to spawn after one winter in the ocean (or 1SW salmon), and (2) the “non‐maturing component” that includes salmon that remain in the ocean for at least two winters. Because density‐dependent effects are more likely to occur among individuals of a similar size, we developed estimates of size‐specific population abundance (Andersen et al., 2017). For salmon in the first winter at sea, we assumed that growth would be affected by the abundance of North American salmon in the same smolt cohort; we assume that salmon from the European stocks have not yet migrated to the Northwest Atlantic in their first year. Therefore, we summed the PFA for the maturing and non‐maturing components from North America by smolt cohort since 1971. For salmon in the second winter at sea, we assumed that growth would be affected by salmon from the NAC and SNEAC, which are both present in the Labrador Sea and waters around Greenland, Iceland, and the Faroe Islands during their second year at sea. Therefore, we summed the PFA of the non‐maturing salmon from the NAC and SNEAC for each year since 1972 (ICES, 2023).

#### 2.3.5 Commercial fisheries harvest

Size‐selective fisheries or fisheries that exploit specific age groups can contribute to observed changes in body size (Law, 2000). For Atlantic salmon in eastern Canada, we focused on the mixed‐stock marine fisheries at West Greenland, Newfoundland and Labrador, and Saint Pierre and Miquelon that harvest salmon from stocks throughout Eastern Canada and could drive synchronous changes in body length. Since 1998, there has been an export ban on salmon caught in the commercial fishery at West Greenland and the exploitation rates have declined (ICES, 2023); this fishery harvests salmon from both North American and European stocks (Bradbury et al., 2021). The commercial fishery in Labrador closed in 1998, and since that time salmon have been harvested by Indigenous Food, Social and Ceremonial fisheries and in the subsistence food fisheries of Labrador residents. The commercial fishery in Newfoundland closed in 1992 and no marine salmon fisheries have been allowed since then. Prior to the closures, commercial fisheries in Newfoundland and Labrador harvested salmon from all regions of eastern North America (Miller et al., 2012; Ritter, 1989), but since the closures, >95% of the salmon harvested in the Labrador subsistence salmon fisheries were from rivers in Labrador (Bradbury et al., 2015; ICES, 2021). Commercial and recreational marine fisheries for Atlantic salmon in Saint Pierre and Miquelon (France's territorial waters) are also mixed stock fisheries that harvest salmon from most stocks of eastern North America (Bradbury et al., 2016; ICES, 2023).

To measure the effects of these fisheries on Atlantic salmon body size, we calculated the exploitation rates for different sized salmon using data from ICES (2023). First, as the West Greenland fishery mostly harvests salmon during their second year at sea (i.e., individuals that did not mature after the first year and are likely to mature as 2SW), the West Greenland exploitation rate was defined as the proportion of total number of salmon from the North American Commission (NAC) caught at West Greenland in relation to the NAC pre‐fishery abundance of 1SW non‐maturing salmon. Second, salmon captured in fisheries around Newfoundland and Labrador, and Saint Pierre and Miquelon are expected to be on their return migration to spawn. An overall exploitation rate for these three regions was calculated for small and large salmon, that is, predominately mature 1SW and 2SW, respectively. This exploitation rate was the proportion of the summed catches across all three fisheries relative to the summed returns across the NAC area, hereafter referred to the exploitation rate in NAC fisheries. Area‐specific exploitation rates for the NAC and West Greenland fisheries are shown in Figure S6.

### 2.4 Statistical analysis

We undertook a two‐step approach to investigate changes in body length for the time‐series and rivers considered. First, we evaluated spatial synchrony in mean fork length trends among rivers using a dynamic factor analysis (DFA). Then, we incorporated covariates to the best‐fitting DFA from the first step to determine the relationships between mean fork length and climate, thermal habitat, food availability, fisheries harvest, and density dependence. All analyses were performed in R version 4.2.2 (R Core Team, 2022), and specific packages used for each step of the analysis are indicated in the sections below.

#### 2.4.1 Synchrony in fork length changes

To determine if there are common trends among rivers in mean annual fork length (mm) over time, we performed a dynamic factor analysis (DFA). DFA has been extensively used in the fisheries literature to investigate questions about spatial synchrony across populations (e.g., Bordeleau et al., 2019; Malick et al., 2023; Mills et al., 2013; Ohlberger et al., 2018). DFA is a dimension‐reduction approach used with timeseries data to identify the smallest number of possible common trends among timeseries (Zuur, Tuck, et al., 2003). DFA is applicable in time series that may contain missing values (Zuur, Fryer, et al., 2003). In its simplest form, DFA models can be specified as: *y*_*t*_ = *Zx*_*t*_ + *a* + *v*_*t*_ where *v*_*t*_ ~ MVN(0, *R*) and *x*_*t*_ = *x*_*t*–1_ + *w*_*t*_ where *w*_*t*_ ~ MVN(0, *Q*) (Zuur, Fryer, et al., 2003; Zuur, Tuck, et al., 2003). Specifically, *y*_*t*_ is modeled as the product of the loading(s) (*Z*) by the common trend(s) (*x*_*t*_) plus offsets (a) and observation error (*v*_*t*_), and the common trend(s) (*x*_*t*_) are modeled with a random walk from *x*_*t*–1_ plus latent error or “noise” (*w*_*t*_). For the full mathematical formation of these models, see Zuur, Fryer, et al. (2003), Zuur, Tuck, et al. (2003). For each river or timeseries, values for mean annual fork length were centered around the river‐specific mean.

For each age class, we ran 12 DFA models using the R package MARSS which implements DFA in a state‐space framework (Holmes et al., 2012, 2021). Similar to other dimension‐reducing approaches (such as PCA), the maximum possible number of common trends in a DFA is one less than the total number of timeseries (Zuur, Fryer, et al., 2003; Zuur, Tuck, et al., 2003); however, most analyses include a much smaller number of trends relative to the number of timeseries (e.g., 1–3 trends for 11 timeseries, Bordeleau et al. (2019); 1–4 trends for nine timeseries, Malick et al. (2023); one trend for five timeseries, Ohlberger et al. (2018)). As negative loadings indicate the inverse pattern of the common trend, the total number of different trends across all timeseries is twice the number of common trends specified in the analysis. Our models included all combinations of 1–4 common trends and one of three variance–covariance structures (*R*) for the observation error (*e*_*t*_): (1) “diagonal and equal” which assumes that timeseries have similar variance, but no covariance between timeseries; (2) “equalvarcov” which assumes that the variance and covariance between timeseries are the same; and (3) “diagonal and unequal” which assumes that each timeseries has different variance and do not co‐vary. In the preliminary analysis, we attempted to use “unconstrained” option which assumes that the variance and covariance are different between rivers. However, this structure requires the most data and none of these models converged, potentially due to the large number of missing values for some rivers (Holmes et al., 2021); thus, it was not included in the results. Additionally, as we lacked a priori information about the spatial structure across Eastern Canada, we did not specify a novel variance–covariance structure. Our models were set to run with a maximum number of iterations of 1,000,000; all but two models during the 2SW analysis converged, and the models that did not converge were excluded from further consideration. In these models, we did not include any covariates as we were interested in examining the synchrony in fork length trends across timeseries.

To determine the best‐fit model, we first compared the models for each age class using Akaike's information criterion with the small sample size correction (AIC_c_). When two or more models were within two AIC_c_, we first considered whether any models had a common trend that was unimportant (e.g., small loadings) as an indication that too many common trends were being modeled (Zuur et al., 2007). If this was not the case, then we also considered which model had fewer parameters (i.e., smaller number of trends and simpler variance–covariance structure); models with fewer trends have fewer parameters, are less complex and are preferred over more complex models (Bordeleau et al., 2019; Zuur, Tuck, et al., 2003). We also considered log likelihood values during model selection, and identified whether the top model(s) had the highest log likelihood. Finally, we inspected QQ‐plots, plots of residuals versus fitted values, and autocorrelation plots (Zuur et al., 2007). Once we determined the final model for each age class, we performed a varimax rotation on the factor loadings and common trends to enhance the interpretation of the model results (i.e., maximize difference between loadings) (Zuur, Fryer, et al., 2003). For each river, the common trends with the highest absolute loading were more important to the overall river‐specific trend than the common trends with lower loadings.

To determine the degree of synchrony across rivers throughout Eastern Canada, we considered the number of common trends in the best‐fit model, the overall shape of the common trends, and the magnitude and direction of the loadings. For example, a DFA model indicating a high degree of spatial synchrony throughout Eastern Canada would include a single common trend and all rivers would have a high loading in the same direction. In contrast, a larger number of common trends would suggest weaker synchrony, particularly if there were no shared features in the shape of these trends (e.g., one trend increases through time, another trend was flat, etc.) and there were no discernable spatial patterns in the magnitude or direction of the loadings. Lastly, support for weak spatial synchrony could include shared features in the shape of multiple common trends and/or regional variation in the magnitude and/or direction of the loadings.

When the best‐fit DFA model included more than one common trend (see Section 3), we performed an agglomerative hierarchical cluster analysis (AHCA) on the loadings with the R packages cluster (Maechler et al., 2022) and factoextra (Kassambara & Mundt, 2020) as a supplemental approach to determine spatial synchrony. The loadings were centered and scaled (mean = 0, SD = 1) and the analysis used the complete linkage method which finds the maximum distance between points belonging to different clusters. Lastly, for these loadings, we calculated the gap statistic to determine the optimal number of clusters over a range of clusters from one to six (or 2 × the number of loadings).

#### 2.4.2 Significance of fork length changes

DFA models do not determine whether there are significant changes in timeseries values. Therefore, we identified periods when the 95% confidence intervals (CIs) for the resulting trend for each river did not span zero. If there were at least two periods when the 95% CIs were entirely above and below the mean river‐specific fork length, then we considered the change to be significant; otherwise we lacked sufficient evidence to identify significant changes. This approach likely underestimates the true number of significant changes in the dataset, particularly for rivers with less data and broader confidence intervals. Therefore, to confirm whether the observed changes in fork length from the DFA model were significant, we also modeled the change in mean annual fork length (centered around the river‐specific mean) for each river with a generalized additive model (GAM) using the R package mgcv (Wood, 2017) during periods (~20 years) of the timeseries when the common trend(s) indicated large changes in fork length. These GAMs solely included rivers with at least 10 years of data during the period being modeled. Full details on this approach and results are included in the Supporting Information. Although these approaches are not directly equivalent, we considered that there was a significant change in fork length if it was documented in either the DFA or GAM.

#### 2.4.3 Covariates and fork length

After determining the best‐fitting model (i.e., number of common trends and variance–covariance matrix) for the relationship between fork length in 1SW and 2SW salmon over time, we re‐ran the best model with additional terms (*Dd*_*t*_), that is *y*_*t*_ = *Zx*_*t*_ + *a* + *Dd*_*t*_ + *v*_*t*_, where *D* is the river‐specific regression coefficients and *d*_*t*_ is the timeseries values for each covariate (Zuur, Fryer, et al., 2003; Zuur, Tuck, et al., 2003). For 1SW salmon, we included covariates that were only related to conditions experienced in the first year at sea, while for 2SW salmon, we included covariates for conditions in both the first and second years at sea (Table 1). Due to the large number of correlated variables (Tables S4 and S5, and Figures S8 and S9), we ultimately decided to consider each covariate independently, rather than determine the best combination of covariates. Therefore, we re‐ran the best‐model (see Section 3), hereafter the “base model,” with each of the eight covariates for 1SW and 15 covariates for 2SW salmon; covariates were centered and scaled.

We identified any covariates that improved on the base model. Conventional model selection criteria indicate that models are preferred when the ΔAIC_c_ is lower than 2 × the number of additional parameters (Arnold, 2010); for DFA models, each covariate results in one additional parameter/timeseries (i.e., river in our analysis) (Zuur, Tuck, et al., 2003). Applying this criterion to our models indicates that for a single covariate to improve on the base model, the ΔAIC_c_ would need to be >38 for 1SW and 16 for 2SW salmon. However, Zuur, Tuck, et al. (2003) showed that DFA models with covariates may not perform better (based on AIC) than models without covariates when the covariate is only related to a few timeseries. Further, this does not necessarily mean the covariate is unimportant (Zuur, Tuck, et al., 2003). The data for some rivers in our analysis contained missing values for many years (Figure 2) spanning periods with large changes in some covariate values. For example, we solely had data for the English River from 1999 to 2019; this period does not span the full range of values for many covariates (e.g., the negative phase of AMO prior to ~1995 or high marine abundance and exploitation rates in the 1970s and 1980s). Therefore, for rivers with an incomplete dataset, it may be challenging to determine relationships with some covariates. As our covariates all had a strong biological rationale for their inclusion, we chose to identify any covariates that improved on the base model (i.e., lower AIC_c_ value and higher log likelihood). We determined if any of the covariates had a significant effect on mean fork length for 1SW and 2SW salmon by summarizing the regression coefficients and corresponding 95% confidence intervals for each covariate and river. If the confidence interval did not span zero, we determined that the covariate had a significant effect on fork length (Zuur, Fryer, et al., 2003). Model fits for the DFA models with covariates that had the lowest AIC_c_ (see Section 3) are provided in Figures S10 and S11.

## 3 RESULTS

**FIGURE 5:**
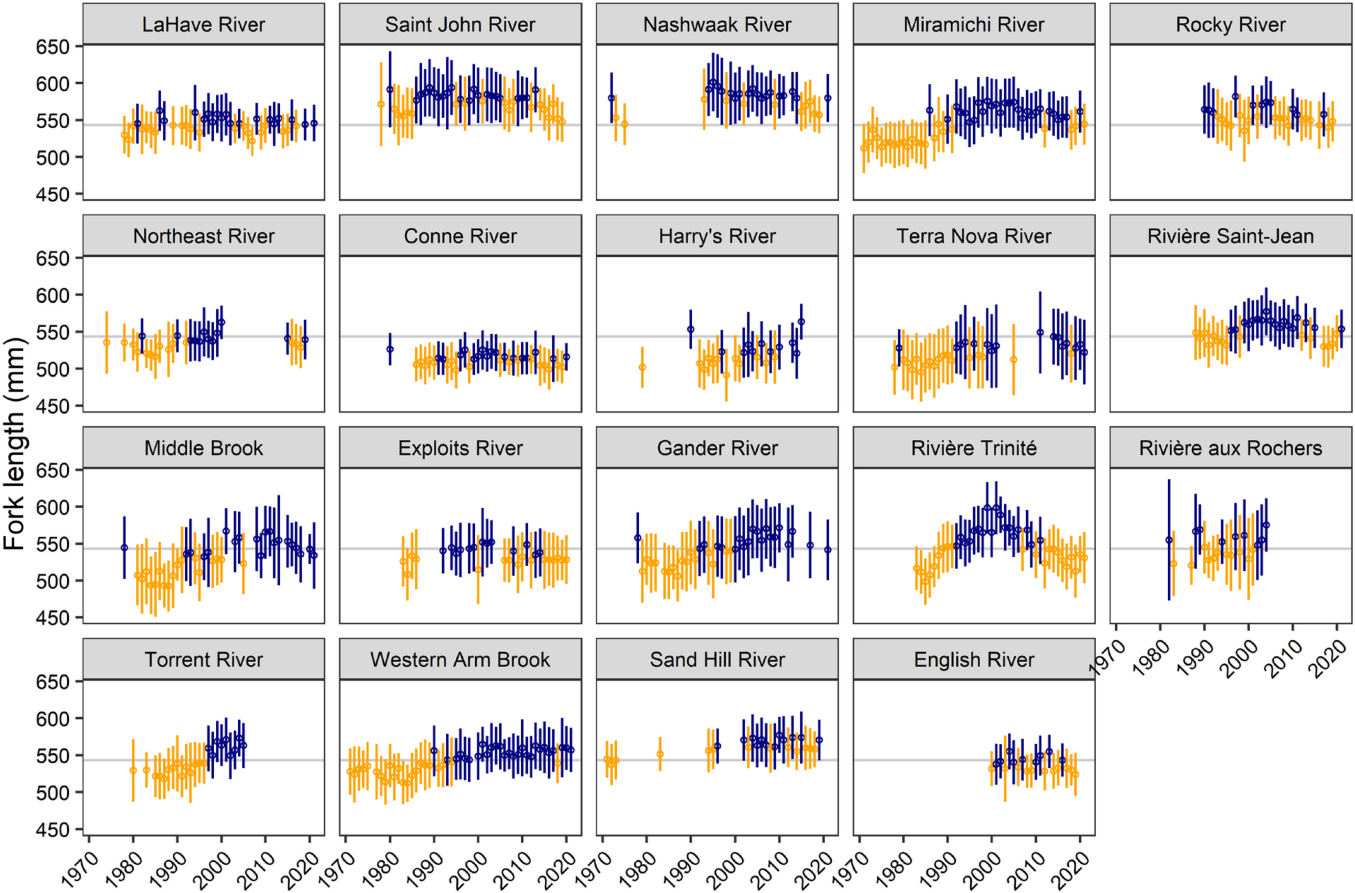
Mean ± one standard deviation in 1SW Atlantic salmon fork length from 1971 to 2021 for 19 rivers across Eastern Canada. Rivers are arranged in order of increasing latitude (top left to bottom right). The colors indicate when the mean was below (orange) or above (blue) the annual mean fork length of each river and the gray line indicates the mean fork length for 1SW salmon across all rivers and years.

**FIGURE 6:**
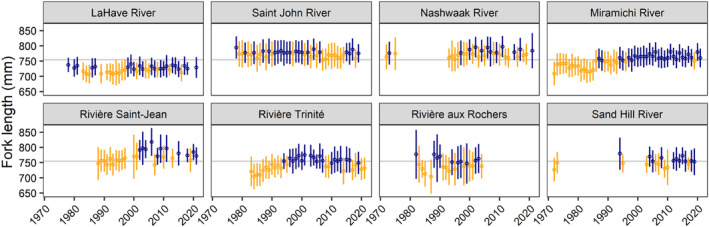
Mean ± one standard deviation in 2SW Atlantic salmon fork length from 1972 to 2021 for eight rivers across Eastern Canada. Rivers are arranged in order of increasing latitude (top left to bottom right). The colors indicate when the mean was below (orange) or above (blue) the annual mean fork length of each river and the gray line indicates the mean fork length for 2SW salmon across all rivers and years.

The mean fork lengths of 1SW and 2SW Atlantic salmon throughout Eastern Canada varied by river across all five decades (Figures 5 and 6). Across all rivers and years, the mean fork length was 543 ± 17 mm for 1SW and 754 ± 18 mm for 2SW; we refer to this as the combined mean. For 1SW salmon from rivers in insular Newfoundland, the mean fork length was generally less than the combined mean.

For the eight rivers where we have data for 1SW and 2SW salmon, there were differences and similarities in body length through time. For LaHave, Saint John, Nashwaak, and Sand Hill, the mean annual fork length for 1SW and 2SW salmon varied without apparent temporal trend through the timeseries. For the Miramichi, 1SW and 2SW salmon had a smaller mean fork length for their age class in the first two decades, after which fork length was longer than the mean in most years. For the Saint‐Jean, 1SW and 2SW salmon generally had longer annual mean fork lengths after ~2000. Lastly, for the Trinité, 1SW and 2SW salmon had longer annual mean fork lengths in the mid‐1990s and 2000s.

### 3.1 Synchrony in fork length changes

#### 3.1.1 1SW salmon

**FIGURE 7:**
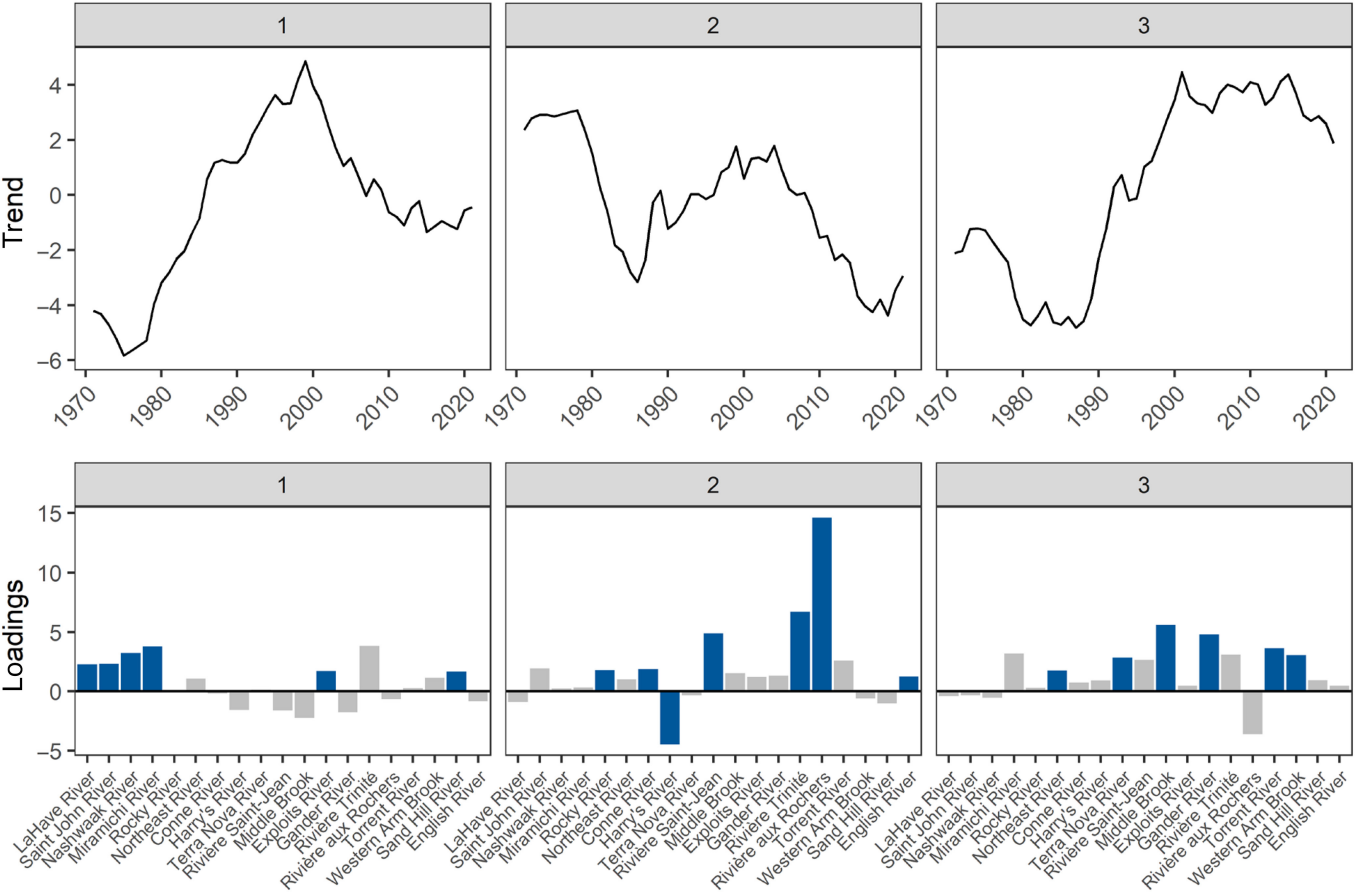
The three common trends and loadings for the dynamic factor analysis that examined changes in 1SW Atlantic salmon fork length from 1971 to 2021 throughout Eastern Canada without accounting for covariates (base model). Rivers are arranged in order of increasing latitude. Trends and loadings are dimension‐less, and blue bars represent the highest loading across trends for each river.

For 1SW Atlantic salmon from 1971 to 2021, the best‐fitting model with the lowest AIC_c_ had three common trends with an equal variance and covariance structure (“equalvarcov”) for observation error (Table 2); this model also had one of the higher log likelihood values (Table 2). Rivers had both positive and negative loadings, the latter indicating that the inverse trend was observed for that river (Figure 7). The first common trend characterized an increase from the mid‐1970s until the highest value in 1999, before declining again. The second common trend was initially stable before rapidly declining from the late 1970s to 1986, then increasing until the late 1990s, and finally declining again after 2004. The third common trend decreased until the late 1970s and was relatively stable during the 1980s and then increased rapidly until the early 2000s and remained stable for the next two decades.

**TABLE 2:** Model comparison based on AIC_c_ for the dynamic factor models without covariates for 1SW and 2SW Atlantic salmon from distinct rivers and runs in Eastern Canada. The top model for 1SW and 2SW salmon is bolded. Only the top five models for each age class are shown; the full model comparison table for the 12 models examined per age class is provided in Table S6.

	# of common trends	Variance–covariance structure	ΔAIC_c_ ^a^	AIC_c_ weight	Log likelihood
1SW	**3**	**equalvarcov**	**0**	**0.91**	**−2260.93**
	4	equalvarcov	4.73	0.09	−2243.37
	2	equalvarcov	26.25	<0.01	−2294.03
	4	diagonal and equal	33.79	<0.01	−2259.17
	3	diagonal and unequal	36.05	<0.01	−2257.74
2SW	**1**	**diagonal and unequal**	**0**	**0.51**	**−1036.83**
	3	equalvarcov	1.89	0.20	−1029.59
	3	diagonal and unequal	2.14	0.17	−1022.32
	3	diagonal and equal	4.50	0.05	−1032.09
	2	diagonal and unequal	4.69	0.05	−1030.99

^a^ AIC_c_ values for the 1SW and 2SW models were 4809.14 and 2123.51, respectively.

**FIGURE 8:**
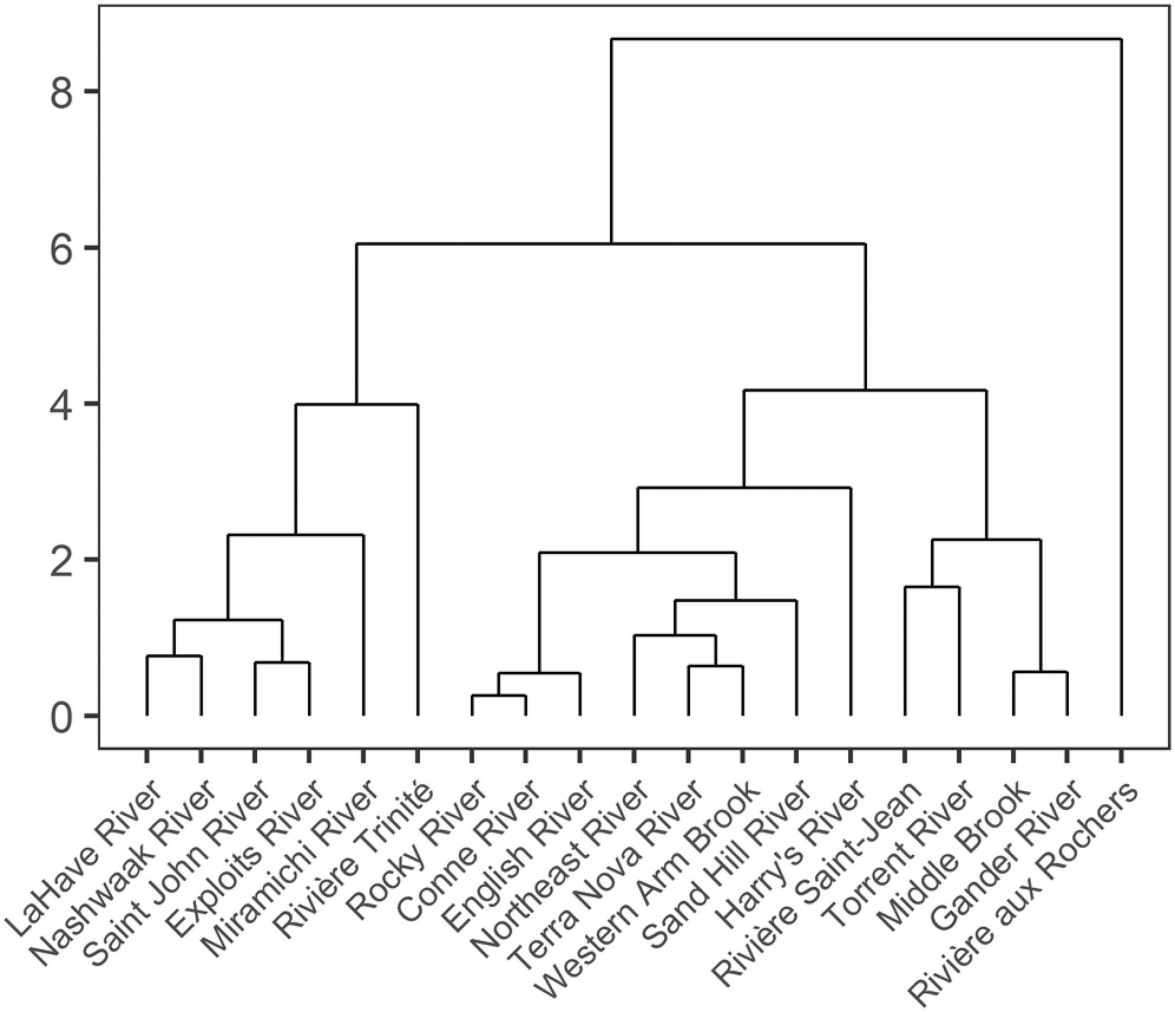
Dendrogram showing the results of an agglomerative hierarchical cluster analysis (AHCA) on the loadings from the best‐fitting dynamic factor analysis (DFA) for 1SW Atlantic salmon fork length from 1971 to 2021 throughout Eastern Canada. The gap statistic indicated that a single cluster was optimal.

**FIGURE 9:**
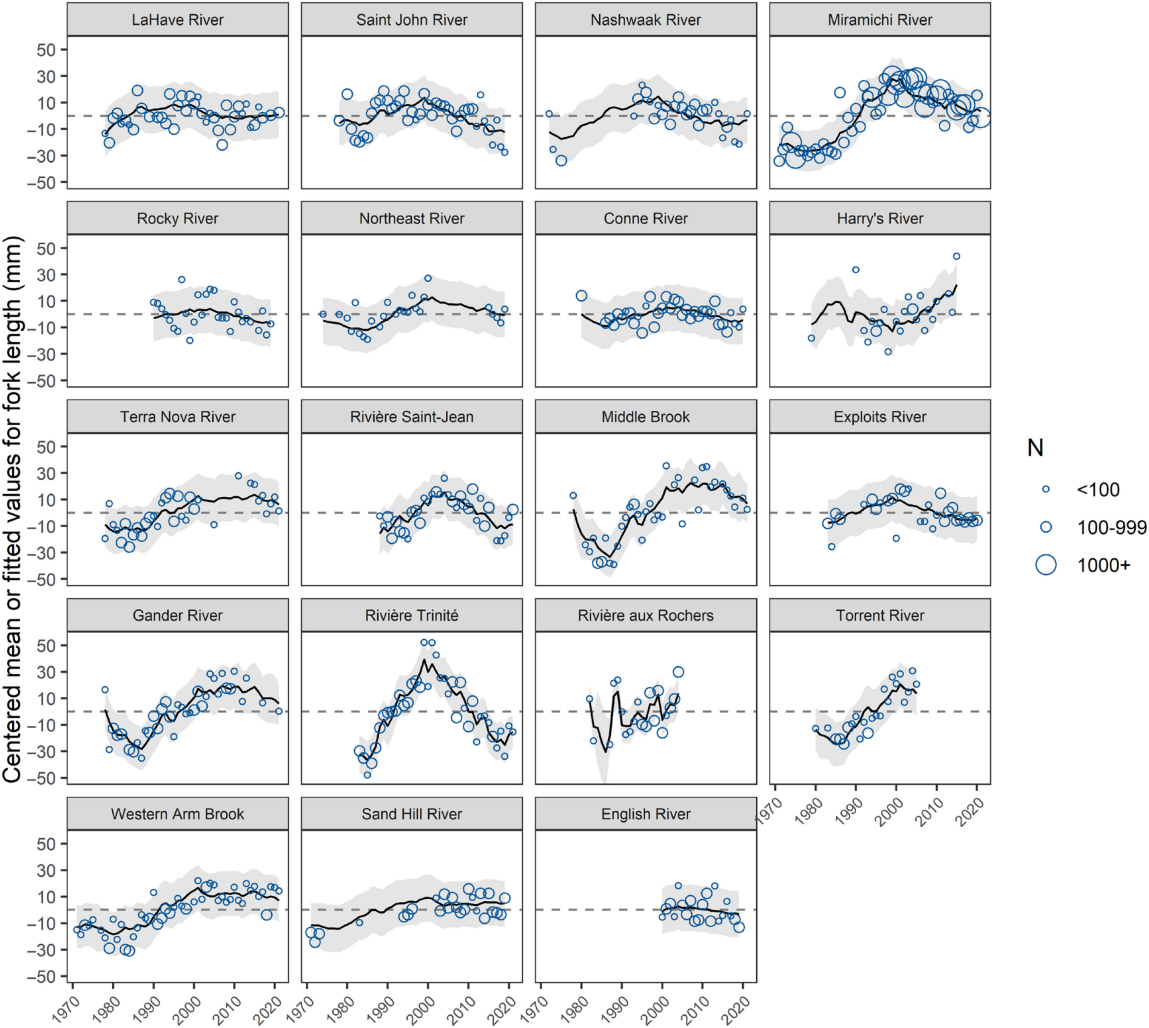
DFA predicted values ±95% CIs for 1SW Atlantic salmon fork length from 1971 to 2020 for rivers across Eastern Canada without accounting for covariates (base model). The blue points represent the mean fork length for each timeseries centered around the river‐specific mean when data were available and the size of these points reflects the sample size used to calculate the mean (N). Rivers are arranged in order of increasing latitude from top left to bottom right.

Most rivers had relevant loadings with absolute values that exceeded one (max. loading value 14.61; Table S7) for two or three common trends indicating that their individual patterns of change were a representation of two or more of the common trends (Figure 7, Table S7). This was confirmed by the AHCA which showed shallow divisions among the rivers (Figure 8) and the gap statistic indicated a single optimal cluster from a range of one to six clusters. The highest DFA loadings for the three most southern rivers that discharge into the Bay of Fundy (Saint John and Nashwaak) or Atlantic Ocean around southern Nova Scotia (LaHave) were for the first trend, but there was no significant change (i.e., confidence intervals spanned zero) in fork length over time (Figure 9). There were three additional rivers that had their highest loadings for the first trend (Figure 7). The Miramichi River, which discharges into the Gulf of St. Lawrence, also had a high loading for the third trend, and overall, there was a significant increase of ~55 mm or ~11% (from the predicted values) in the fork length of 1SW salmon during the 1980s and 1990s. A significant increase in fork length for the Miramichi was also found in the GAM (Table S9, Figure S13). The other two rivers, Exploits and Sand Hill that discharge from the northeastern coast of Newfoundland and coast of Labrador, respectively, had confidence intervals for their DFA trends that included zero; however, the GAM showed a significant increase in fork length for the Exploits. Except for Sand Hill, all of these rivers and Trinité were clustered together by the AHCA (Figure 8).

The remaining mid‐latitude and northern rivers had their highest loadings for the second and third trends (Figure 7). Most rivers that discharge into the southern Gulf of Saint Lawrence (Harry's, Saint‐Jean, Trinité, and aux Rochers) as well as a two of three rivers that discharged into the Atlantic Ocean from the southern coast of Newfoundland (Rocky and Conne) and one of two rivers that discharge into the Labrador Sea (English) had their strongest loadings for the second trend. Only the Trinité and aux Rochers had significant increases of ~76 and ~43 mm (~15% and ~9%), respectively, in body size during the 1980s and 1990s. There was also a significant decrease of ~51 mm (~9%) in fork length for the Trinité during the 2000s and 2010s (Figure 9). Significant changes in fork length for the Trinité were also confirmed by the GAM (Table S9, Figure S13). Rivers with the highest loading on the third common trend included the remaining river that discharged into the Atlantic Ocean from the southern coast of Newfoundland (Northeast) as well as most rivers from the northeastern coast of Newfoundland (Middle Brook and Gander), and two rivers that discharge into the northern Gulf of St. Lawrence (Torrent and Western Arm Brook) (Figure 7). Of these rivers, Middle Brook, Gander, and Torrent had a significant increase in fork length of ~53, ~46, and ~45 mm (11%, 9%, and 9%), respectively, during the 1980s and 1990s (Figure 9). Significant increases in fork length for this period were also confirmed with the GAM for these rivers, as well as Northeast and Western Arm Brook (Table S9, Figure S13). With the exception of aux Roches and Trinité, all of these rivers and Sand Hill were clustered together by the AHCA; aux Rochers was not clustered with any other rivers (Figure 8).

#### 3.1.2 2SW salmon

**FIGURE 10:**
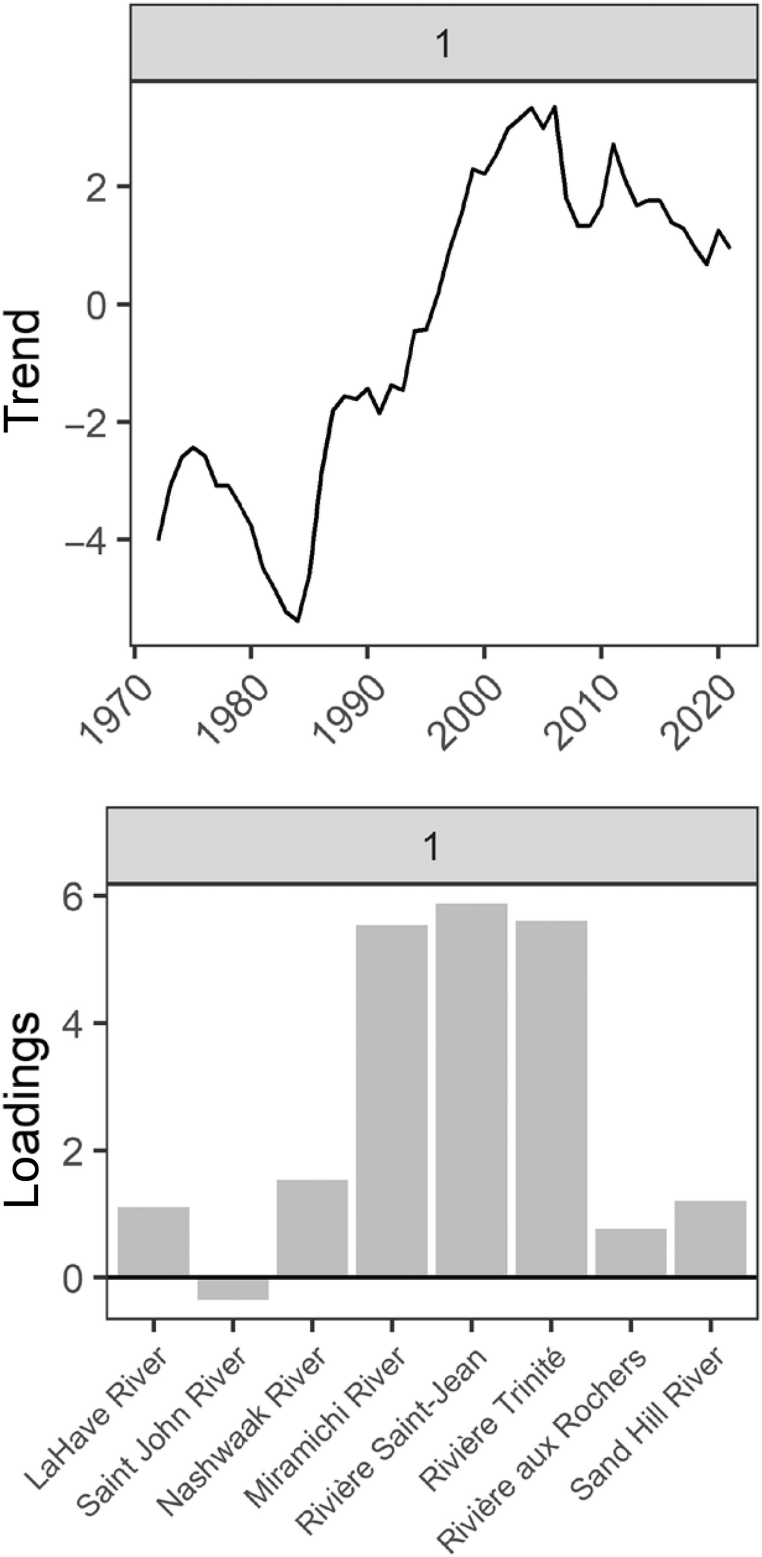
The common trend and loadings for the dynamic factor analysis that examined changes in 2SW Atlantic salmon fork length from 1972 to 2021 throughout Eastern Canada without accounting for covariates (base model). Rivers are arranged in order of increasing latitude. Trends and loadings are dimension‐less.

**FIGURE 11:**
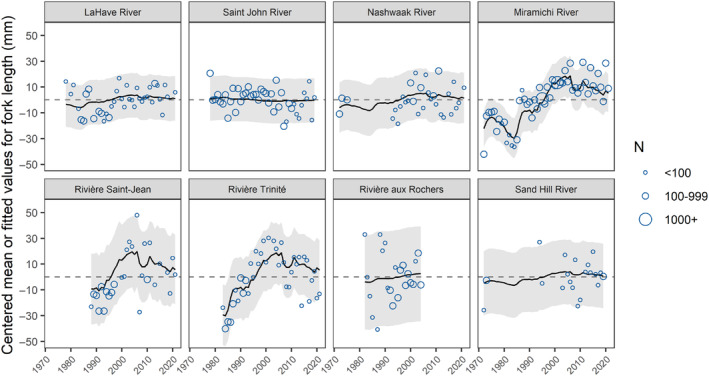
DFA Predicted values ±95% CIs for 2SW Atlantic salmon fork length from 1972 to 2021 for rivers across Eastern Canada without accounting for covariates (base model). The blue points represent the mean fork length for each timeseries centered around the river‐specific mean when data were available and the size of these points reflects the sample size used to calculate the mean (N). Rivers are arranged in order of increasing latitude from top left to bottom right.

For 2SW Atlantic salmon from 1972 to 2021, the DFA model with the lowest AIC_c_ value included one common trend and “diagonal and unequal” variance and covariance structure (Table 2). The second best model (ΔAIC_c_ = 1.89) included three common trends and “equalvarcov” variance and covariance structure. However, this model had relatively small loadings for the third common trend (Figure S10). Neither model had the highest log likelihood value. Therefore, we determined the model with the lowest AIC_c_ had the most support. For this model, the single common trend had an overall increasing pattern that leveled off in the most recent years (Figure 10). Loadings for most rivers that discharge into the Gulf of St. Lawrence (Miramichi, Saint‐Jean, and Trinité) were relatively high for this trend (Table S7). In contrast, loadings for the most southern rivers that discharge into the Bay of Fundy (Saint John and Nashwaak), Atlantic Ocean around southern Nova Scotia (LaHave), one river that discharges into the Gulf of St. Lawrence (aux Rochers), and a northern river that discharges into the Labrador Sea (Sand Hill) were much lower. Only the Miramichi River showed a significant increase of ~48 mm or ~7% (from the predicted DFA values) in fork length during the 1980s and 1990s (Figure 11). However, both the Miramichi and Trinité had a significant increase in fork length in the GAM (Table S9, Figure S14).

### 3.2 Covariates and fork length

#### 3.2.1 1SW salmon

**FIGURE 12:**
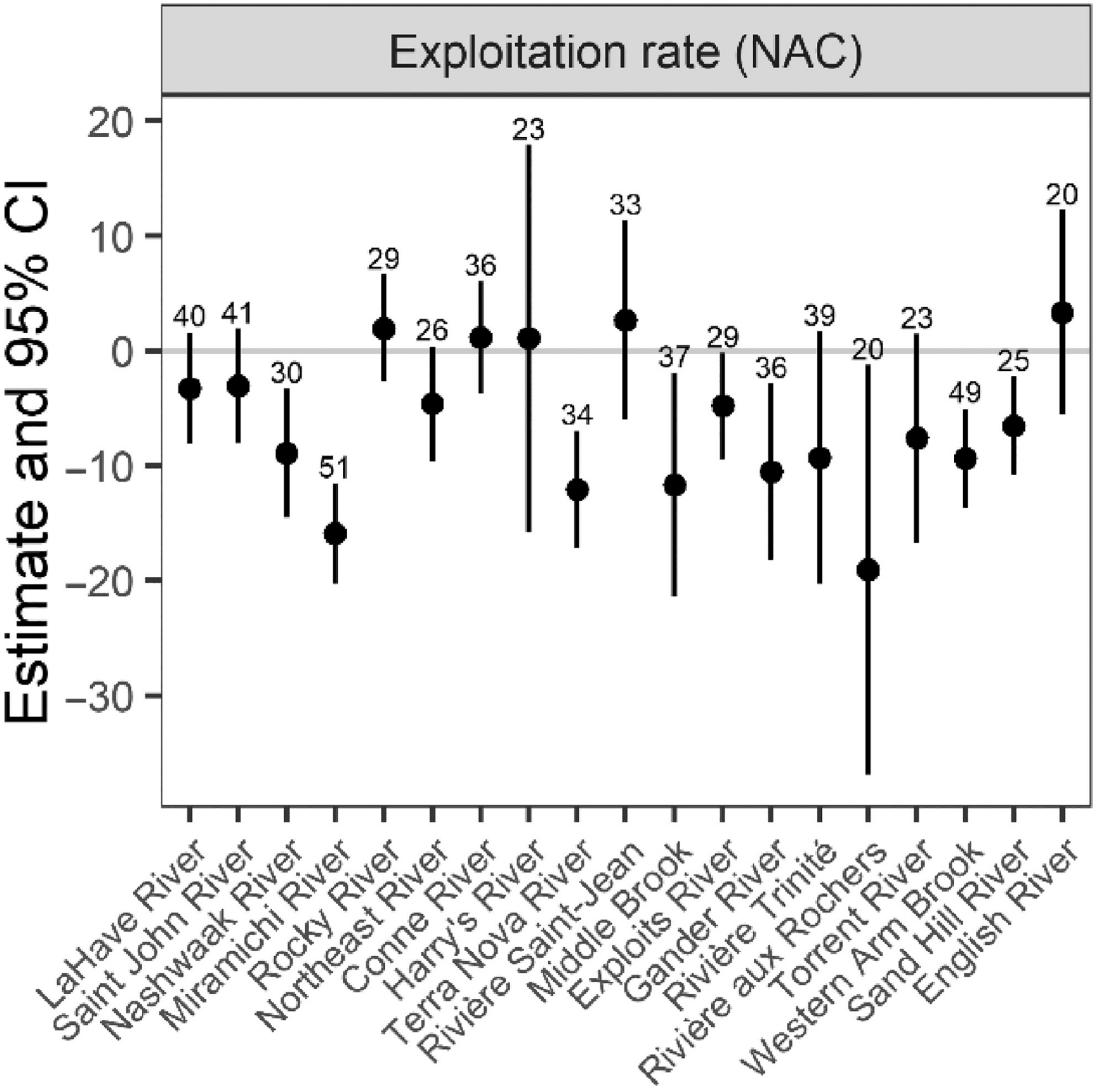
Parameter estimate (±95% CIs) for the relationship between the exploitation rate for small Atlantic salmon in the Newfoundland and Labrador, and St. Pierre and Miquelon fisheries and the fork length of 1SW salmon for 19 rivers along a latitudinal gradient throughout Eastern Canada. Rivers are arranged in order of increasing latitude, and the number of years of data for each river is shown.

For 1SW salmon from 1971 to 2021, one model had a lower AIC_c_ and higher log likelihood than the base model without covariates. The model with the lowest AIC_c_ solely included the exploitation rate for small salmon in NAC fisheries (ΔAIC_c_ = −9.74; Table 3). Exploitation rates had a significant negative effect on mean fork length for salmon in nine rivers (in order of increasing latitude: Nashwaak, Miramichi, Terra Nova, Middle Brook, Exploits, Gander, aux Rochers, Western Arm Brook, and Sand Hill) across much of the latitudinal gradient (Figure 12). The remaining rivers had confidence intervals that spanned zero, so we did not conclude that there was a statistically significant relationship.

**TABLE 3:** Model selection table for the relationships between climate indices, thermal habitat area, food availability, density dependence, and fisheries harvest and the body size of Atlantic salmon in Eastern Canada from 1971 to 2021 (1SW) or 1972 to 2021 (2SW). Only the models that improved on the base model described in the previous section are shown (i.e., models with a lower AIC_c_ than the model without covariates); the full model comparison table is provided in Table S8.

	Model^a^	ΔAIC_c_ ^b^	logLik
1SW	Exploitation rate (NAC)	−9.74	−2232.26
	Base (no covariates)	0	−2260.93
2SW	Winter thermal habitat_2_	−12.27	−1021.30
	Exploitation rate (NAC)	−5.06	−1024.91
	AMO_2_	−5.03	−1024.93
	NLCI_2_	−4.31	−1025.29
	NLCI_1_	−3.51	−1025.69
	NAO_2_	−1.53	−1026.68
	Base (no covariates)	0	−1036.83

Abbreviations: NAC, North American Commission includes salmon harvested in Newfoundland and Labrador, and St. Pierre and Miquelon fisheries; AMO, Atlantic Multidecadal Oscillation; NAO, North Atlantic Oscillation; NLCI, Newfoundland and Labrador Climate Index.

^a^ Subscripts for 2SW covariates: Numbers denote the 1st or 2nd year at sea.

^b^ ΔAIC_c_ values represent the difference in AIC_c_ between the base model and each model with covariates. They are expressed as a negative value to show improvements in the base model but are not considered to be the best model.

#### 3.2.2 2SW salmon

**FIGURE 13:**
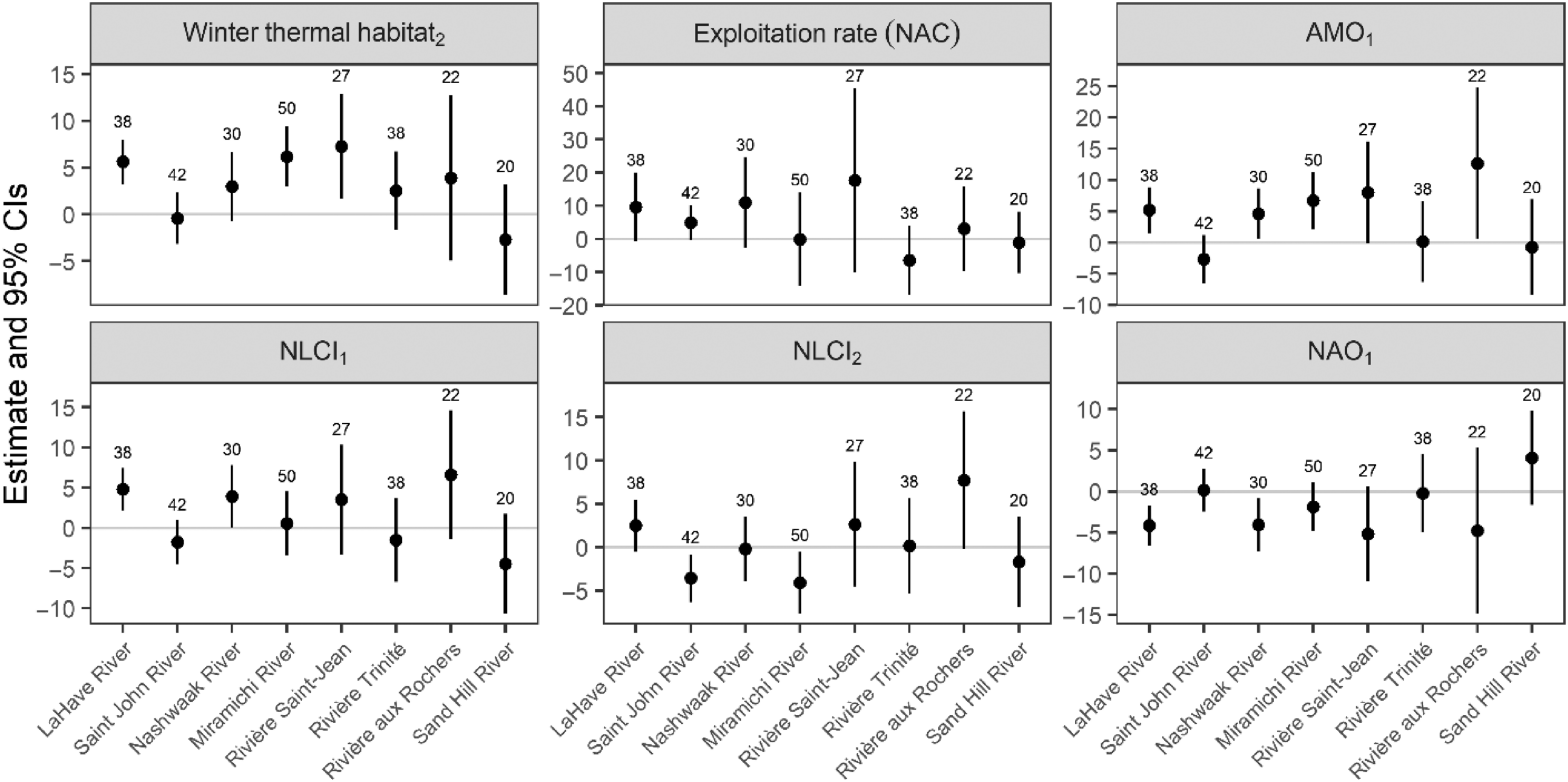
Parameter estimate (±95% CIs) for the relationships between winter thermal habitat in the second year, exploitation rate for salmon in NAC fisheries, and climate indices (AMO in the second year, NLCI in both years, and NAO in the second year) and the fork length of 2SW Atlantic salmon for eight rivers along a latitudinal gradient throughout Eastern Canada. Rivers are arranged in order of increasing latitude, and the number of years of data for each river is shown. Covariates are arranged in order of the greatest (winter thermal habitat_2_) to least (NAO_2_) improvement in AIC_c_ from the base model. Subscripts indicate the year of the covariate or specific fisheries. AMO, Atlantic Multidecadal Oscillation; NAC, North American Commission and includes salmon harvested in Newfoundland and Labrador, and St. Pierre and Miquelon fisheries; NAO, North Atlantic Oscillation; NLCI, Newfoundland and Labrador Climate Index.

For 2SW salmon from 1972 to 2021, there were six models with lower AIC_c_ values and higher log likelihood than the base model (Table 3). The addition of winter thermal habitat in the second year resulted in the greatest improvement on AIC_c_ (ΔAIC_c_ = −12.27). There was a significant, positive relationship between the area of winter thermal habitat and fork length for three rivers (LaHave, Miramichi, and Saint‐Jean) (Figure 13). The next model with the greatest improvement in AIC_c_ (ΔAIC_c_ = −5.06) included exploitation rates from NAC fisheries, but we were unable to determine the direction of these relationships for any rivers (i.e., confidence intervals spanned zero). Third, there was a significant, positive relationship between AMO during the first year and fork length for four rivers (LaHave, Nashwawk, Miramichi, and aux Rochers) (ΔAIC_c_ = −5.03). There was a significant, positive relationship between NLCI during the first year for the LaHave River (ΔAIC_c_ = −4.31), and a significant, negative relationship between NLCI during the second year for the Saint John and Miramichi Rivers (ΔAIC_c_ = −3.51). Finally, there was a negative relationship between NAO during the first year and fork length for the LaHave and Nashwaak Rivers (ΔAIC_c_ = −1.53).

## 4 DISCUSSION

For 1SW and 2SW salmon in Eastern Canada, we found weak spatial synchrony in mean fork length trends. However, when considered across the latitudinal gradient represented in this study, three distinct groups emerged: (1) southern rivers that discharge into the Bay of Fundy and the Atlantic Ocean around southern Nova Scotia; (2) mid‐latitude rivers that discharge in the southern Gulf of St. Lawrence and Atlantic Ocean around southeastern Newfoundland; and (3) northern rivers that discharge into the northern Gulf of St. Lawrence and Atlantic Ocean around eastern and northern Newfoundland. However, despite the different regional trends, significant changes in fork lengths were only found for salmon from some rivers at mid‐ and northern latitudes. For many rivers, there were increases in fork length ranging from ~38 to 81 mm (or 7%–15%) throughout the 1980s and 1990s, after which fork length either stabilized (northern rivers) or started to decline (mid‐latitude rivers). There was a higher proportion of significant body length changes among 1SW salmon (10/15 rivers with min. 10 years of data during the 1980s and 1990s) compared to 2SW salmon (2/6 rivers); however, five of the rivers included in both analyses, exhibited no significant changes in body length for either age class. Also, for 1SW salmon in one river (Trinité), the increased body length during the 1980s and 1990s was followed by a period of decreasing body length in subsequent decades resulting in a net increase of only ~25 mm. For 1SW salmon, changes in fork length were associated with the exploitation rates of salmon in fisheries around Newfoundland and Labrador, and St. Pierre and Miquelon. Additionally, for 2SW salmon, changes in fork length were also related to winter thermal habitat and several climate indices acting during both years at sea.

### 4.1 Spatial synchrony in fork length changes

Latitudinal gradients in body size trends have been observed in Norwegian Atlantic salmon populations (Vollset et al., 2022); however, among Canadian populations, more pronounced changes in body size occurred at mid‐ and northern latitudes. It is common for the strength of spatial synchrony to decrease with distance or geographic barriers (Liebhold et al., 2004; Walter et al., 2017). In Eastern Canada, it is possible that conditions experienced during migration from rivers to common feeding areas act as a desynchronizing factor (Quinn et al., 2022). For example, southern rivers have the longest migrations as they travel through the Bay of Fundy, Gulf of Maine and/or Atlantic Ocean to reach feeding areas, whereas mid‐latitude populations from the Gulf of St. Lawrence migrate northward through the Strait of Belle Isle (between Newfoundland and Quebec), and northern populations essentially enter the feeding area directly after leaving their rivers (Bradbury et al., 2021; Chaput et al., 2019; Lacroix, 2013). Further, while trans‐oceanic migration has been confirmed (Bradbury et al., 2021), it is still unclear whether the proportions of trans‐oceanic migrants from rivers throughout Eastern Canada are similar. Certainly, the broader area used by salmon during the second year at sea provides an opportunity for regional factors or use of distinct habitats throughout the North Atlantic Ocean to affect trends in the body size of 2SW salmon. Consideration of local (e.g., river‐specific freshwater conditions during juvenile growth) or regional factors, in addition to the shared conditions considered in this study, may be helpful in understanding trends in body size (Tirronen et al., 2022). These factors may also help to explain conflicting findings on the degree of synchrony among other life‐history processes, such as survival (e.g., Olmos et al., 2020; Pardo et al., 2021; Tirronen et al., 2022).

### 4.2 Effects of covariates

We found that higher exploitation rates from marine fisheries around Newfoundland and Labrador and St. Pierre and Miquelon were associated with reduced fork length for 1SW Atlantic salmon returning to nine rivers across the latitudinal range examined. These fisheries use gillnets which can selectively harvest larger individuals (Chadwick & Claytor, 1990; Reddin, 1984). The inverse relationship between fisheries harvest and body size has been documented for Atlantic salmon in North America (Bielak & Power, 1986; Moore et al., 1995) and Europe (Cotter et al., 2022), as well as other marine species globally (Fenberg & Roy, 2008; Ricker, 1981; ter Hofstede & Rijnsdorp, 2011). The selective removal of larger fish may result in a positive feedback relationship that causes further reductions in body size and can impair sustainable harvests (Audzijonyte et al., 2013), slow recovery from overfishing (Birkeland & Dayton, 2005; Olsen et al., 2004) and affect the resilience of populations to other environmental perturbations such as climate change (Planque et al., 2010). Marine exploitation rates are presently at historic lows and have been since the 1990s (Figure S7; ICES, 2023), and therefore, as in Cotter et al. (2022), these fisheries cannot explain the reductions in body size over the last two decades for salmon in some rivers. However, even though present harvest levels are low, the previously high exploitation rates may hinder the recovery of Atlantic salmon populations, possibly as a result of the loss of genetic diversity (Hauser et al., 2002; Law, 2000; Uusi‐Heikkilä et al., 2015, but see Hutchings & Kuparinen, 2020) or changes in the frequency of alleles related to the age when salmon mature (Czorlich et al., 2022).

We found positive relationships between winter thermal habitat and climate indices for 2SW salmon indicating that salmon were longer when temperatures were warmer and habitat had a higher growth potential. However, these effects were generally limited to fewer than half of the rivers monitored and there was little evidence of spatial synchrony in body size for 2SW salmon among these rivers. For five of eight rivers, relationships between fork length and winter thermal habitat in the second year, AMO in the first year, NLCI in the first year, and/or NAO in the second year, suggest that in years when temperatures were warmer and there was less ice, 2SW salmon were longer. In recent years, Atlantic salmon from European populations shows decreased growth and body size associated with warmer temperatures during the first year at sea (Todd et al., 2008, 2021; Vollset et al., 2022). While, our covariates are not directly equivalent, the trends in oceanic thermal habitat and climate indices (Figure 3) indicate that there has been an increase in the amount of suitable habitat to support the growth of salmon from Eastern Canada. Temperatures in the northwest Atlantic Ocean are generally colder than the northeast Atlantic due to the influx of cold water from the Labrador, Irminger, and East Greenland Currents. However, this area is warming (Greene et al., 2008; Häkkinen et al., 2013) and once temperatures reach a critical threshold, further increases in temperature may ultimately result in decreased growth (Handeland et al., 2008). For example, other studies have estimated a 6%–22% decrease in the body size of marine fish for every 1°C of warming (Sheridan & Bickford, 2011). We speculate that the inverse relationship for fork length and NLCI in the second year for two rivers (i.e., shorter fork length in years with warmer conditions with less sea ice and fewer icebergs), may be an indication that at certain times of the year, ocean conditions in the Labrador Sea may be approaching and exceeding these thresholds for Atlantic salmon. For example, the body size of salmon from the Miramichi River is positively related to thermal habitat area in the second winter, but negatively related to NLCI. We modeled the thermal habitat area to increase with temperature up to 15°C, after which it would decline. Therefore, these contrasting findings suggest that temperatures may be at or exceeding a threshold for some Atlantic salmon populations with subsequent declines in body size. An alternative explanation to the relationships between these covariates and body size is food availability. Growth is generally more limited by food availability than temperature (Beauchamp, 2009; Jonsson et al., 2013), and past studies have attributed the mechanism behind the correlations between temperature and growth or body size for Atlantic salmon to changes in food availability (Jonsson et al., 2016; Long et al., 2023; Todd et al., 2008, 2021; Vollset et al., 2022). Incorporating an index of food availability into our models did not result in any improvements to the base model; however, salmon forage on a broad prey field and many common prey items (e.g., shrimp, blue whiting (*Micromesistius poutassou*), sandeel (*Ammodytes tobianus*), herring (*Clupea harengus*), and other mesopelagic fish; Haugland et al., 2006; Jacobsen & Hansen, 2001) were not included in our index of food availability. Therefore, the relationships we observe may reflect the combined effects of temperature and food availability on growth.

We did not find any support for a relationship between marine abundance and body size. This contrasts with the reported density‐dependent effects on marine growth or body size for Atlantic salmon in the Baltic Sea (Huusko & Hyvärinen, 2012), Pacific salmon (Jeffrey et al., 2017; Ohlberger et al., 2023; Oke et al., 2020) and other marine fish (Andersen et al., 2017; Lorenzen & Enberg, 2002; Rindorf et al., 2022; Zimmermann et al., 2018). One plausible reason for our finding is that the abundance of Atlantic salmon in the ocean is not considered to be limiting for growth or survival (Jonsson & Jonsson, 2004a, 2004b) and there is insufficient intra‐species competition for resources to observe a density‐dependent effect. This could explain the contrasting results for density‐dependent effects between Atlantic and Pacific salmon (*Oncorhynchus* spp.), as the abundance of Atlantic salmon has declined by over half since the 1970s (ICES, 2021), whereas the abundance of several sockeye (*O. nerka*), pink (*O. gorbuscha*) and chum salmon (*O. keta*) stocks is at historically high levels due to strong hatchery production (Ruggerone & Irvine, 2018). Regardless of our result, considering density‐dependence as a potential factor in body size or growth changes, even during periods of reduced abundance, is important as changes to the climate, physical, and biological marine conditions may alter the carrying capacity for Atlantic salmon (Beaugrand & Reid, 2003, 2012; Mills et al., 2013).

### 4.3 Limitations, future directions, and management implications

Past studies suggest that Atlantic salmon may follow the growth pattern that started during their first year in the sea (Huusko & Hyvärinen, 2012; Jonsson & Jonsson, 2004a) and that recruitment of 2SW salmon is driven by thermal habitat during the first year at sea (Friedland et al., 1998). However, we found that covariates from both years were related to the final body size of 2SW salmon. These observations are not mutually exclusive, and the period when salmon initially transition to the marine environment (i.e., post‐smolt period) is certainly very important for the survival and growth of young salmon (Friedland et al., 1998; Olmos et al., 2020; Thorstad et al., 2012). However, 2SW salmon abundance has declined more steeply than 1SW, possibly as a result of greater cumulative effects associated with climate and ecosystem conditions during the longer ocean growing period (Mills et al., 2013). For one North American population, growth during the second year at sea is strongly correlated with return rates, and growth in the second year has been decreasing since the 1990s (Barajas et al., 2022). Older, larger salmon have a higher fecundity than smaller, 1SW salmon (Fleming, 1996, 1998), therefore, we suggest that future work should also consider conditions during second and other subsequent years in the marine environment when considering relationships between environmental conditions and growth, body size, or other demographic processes, such as survival and population trends.

Our work has several key limitations that bear mention. First, many covariates were strongly correlated with each other making it difficult to disentangle the direct and indirect effects of each covariate. For example, Beaugrand and Reid (2012), Beaugrand and Reid (2003) demonstrated that warmer temperatures resulted in subsequent changes in zooplankton abundance and a decrease in salmon catches, Mills et al. (2013) found complex relationships across climatic (AMO and NAO) and physical conditions (SST and salinity), zooplankton communities, capelin size, and salmon abundance and productivity. Similarly, Friedland et al. (2014) documented that warmer temperatures (as indicated by AMO) were correlated with lower pre‐fishery abundance. Second, our analyses did not incorporate intra‐year variability in fork length for each river possibly associated with variations in sampling dates; however, because river‐specific sampling periods were relatively consistent across years, we do not expect that this factor would explain the trends reported here. Finally, we lacked many years of data for several timeseries; these missing values may have made it challenging to detect temporal changes in body size and relationships with covariates. For example, it would have been difficult to detect changes in body size for rivers that did not sample or sparsely sampled salmon during the 1980s and 1990s when most changes in body size for the most completely sampled rivers were detected. Similarly, if the river was only sampled during periods of relatively similar environmental conditions, it would have been difficult to detect relationships between body size and covariates. However, at the outset of this work, we were focused on examining trends across the full timeseries and latitudinal gradient, and therefore included as many rivers as possible.

Despite challenges related to data collection for a migratory marine species and practicalities associated with monitoring programs, other biological factors may have influenced our findings and should be the focus of future research. First, spatial synchrony is not a static process and synchronizing factors may strengthen or weaken over time (Liebhold et al., 2004; Walter et al., 2017). Certainly, since the 1970s, there are many broad‐scale factors in the marine ecosystem that are potential synchronizing factors (Beaugrand & Reid, 2003, 2012; Mills et al., 2013; Olmos et al., 2020), many of which have undergone large changes since the 1970s. Therefore, it is possible that the factors driving increased fork length during the 1980s and 1990s are different from the factors that have resulted in more recent declines or stabilizing of fork length changes since the 2000s. The strength of relationships between fork length and different covariates, as well as the interactions between covariates throughout the time‐period is an important area for future work. Second, different conditions experienced during the initial migration to feeding areas and/or differences in the proportion of the salmon from each river undergoing trans‐oceanic migration may explain the different trends observed for rivers across the latitudinal gradient, as well as the relationships between different rivers and the covariates we considered. Third, local river‐specific phenomena, such as physical features of the river and thermal regime (Bradbury et al., 2014; Dionne et al., 2008) may affect the geographic variation in factors like the age and body size of juvenile salmon during marine entry. Throughout Eastern Canada, the number of years spent in freshwater increases with latitude, with the oldest smolts from Labrador, and the youngest smolts from the Maritime provinces and Quebec rivers (Chaput et al., 2006). As marine survival is correlated with smolt body size (e.g., Gregory et al., 2019), then geographic variation in the conditions that affect the age and/or size of salmon smolts may influence populations independently and result in changes that cannot be attributed to shared conditions (Pardo et al., 2021; Tirronen et al., 2022). Finally, while dispersal can act as a synchronizing factor across populations (Bjørnstad et al., 1999; Moran, 1953; Walter et al., 2017), the low straying rates for Atlantic salmon in most wild populations (e.g., 4.4%–15.4%, Keefer & Caudill, 2014), are likely below the threshold (>20%) required for dispersal to drive synchrony (Lamarins et al., 2022). While these considerations were beyond the scope of this study, they are important areas for future research.

The relationship between body size and fecundity is important for fisheries management and species conservation (Audzijonyte et al., 2013; Birkeland & Dayton, 2005). However, although we observed increases in body size for several Atlantic salmon stocks during the 1980s and 1990s, salmon abundance has declined and is presently at less than half of the abundance during the 1970s (Friedland et al., 2014; ICES, 2023). Clearly, the presumed increase in fecundity associated with larger body size would be sufficient to mitigate the reductions in abundance. Concerningly, some of these populations at reduced abundance are showing signs of declining body size which could lead to further population declines. The lack of strong synchrony in recent body size trends, even among geographically close rivers, reinforces the importance of monitoring and managing Atlantic salmon populations at a local and/or regional scale.

## AUTHOR CONTRIBUTIONS

**Tara L. Imlay:** Conceptualization (equal); data curation (equal); formal analysis (lead); methodology (lead); visualization (lead); writing – original draft (lead); writing – review and editing (lead). **Cindy Breau:** Conceptualization (equal); funding acquisition (equal); methodology (equal); supervision (equal); writing – review and editing (equal). **Guillaume J. R. Dauphin:** Conceptualization (equal); funding acquisition (equal); methodology (equal); supervision (equal); writing – review and editing (equal). **Gérald Chaput:** Conceptualization (equal); data curation (equal); methodology (equal); supervision (equal); writing – review and editing (equal). **Julien April:** Data curation (equal); writing – review and editing (equal). **Scott Douglas:** Data curation (equal); writing – review and editing (equal). **J. Derek Hogan:** Data curation (equal); writing – review and editing (equal). **Sherise McWilliam:** Data curation (equal); writing – review and editing (equal). **Daniela Notte:** Data curation (equal); writing – review and editing (equal). **Martha J. Robertson:** Data curation (equal); writing – review and editing (equal). **Andrew Taylor:** Data curation (equal); writing – review and editing (equal). **Kari Underhill:** Data curation (equal); writing – review and editing (equal). **Laura K. Weir:** Conceptualization (equal); methodology (equal); supervision (equal); writing – review and editing (equal).

## CONFLICT OF INTEREST STATEMENT

The authors have no conflict of interest.

## Supporting information


Appendix S1

## Data Availability

Data on the body size of Atlantic salmon and all R code that support the findings of this study are openly available at http://doi.org/10.17605/OSF.IO/78PWT. Covariate data are also either openly available at this location or the locations listed in the Table S2.
