# Supplementary material for: Body length changes for Atlantic salmon (Salmo salar) over five decades exhibit weak spatial synchrony over a broad latitudinal gradient
Source: Ecol Evol. 2024 Jun 9;14(6):e11538. doi: 10.1002/ece3.11538 (PMC11163019; doi:10.1002/ece3.11538)
Supplement: Supplementary file 1 — Appendix S1 [file ECE3-14-e11538-s001.docx]

# Supporting information

## Sampling in Eastern Canadian rivers

Table S1. Summary of the locations, data availability, sampling, and data included in the analyses for Atlantic salmon (*Salmo salar*) rivers in Eastern Canada from 1970-2022, excluding individuals with known or suspected hatchery, captive breeding or smolt-to-adult supplementation program, and aquaculture origins, and an unknown year of sampling. Rivers are arranged in order of increasing latitude.

|  |  |  |  |  |  |  |  |  | Sampling methods | | | | | | | |  |
| --- | --- | --- | --- | --- | --- | --- | --- | --- | --- | --- | --- | --- | --- | --- | --- | --- | --- |
| River | Latitude | Longitude | Total # of years | Min. year | Max. year | Total # salmon | # 1SW salmon (in analysis) | # 2SW salmon (in analysis) | Angling^1^ | Counting fence^2^ | Electrofish^2^ | Fishway^2^ | Gillnet^1^ | Other^3^ | Trapnet^2^ | Unknown | Data source^4^ |
| LaHave River | 44.533 | -64.717 | 41 | 1978 | 2021 | 46,102 | 8,692 | 3,189 |  |  |  | 33,494 |  |  |  |  | 1 |
| Saint John River | 45.955 | -66.608 | 45 | 1978 | 2022 | 266,949 | 12,802 | 10,502 |  |  |  | 179,396 |  |  |  |  | 2 |
| Nashwaak River | 46.118 | -66.589 | 32 | 1972 | 2022 | 13,750 | 3,853 | 2,310 |  | 13,235 |  |  |  |  |  |  | 2 |
| Miramichi River | 46.929 | -65.667 | 51 | 1971 | 2021 | 156,178 | 48,867 | 22,586 |  |  |  |  |  |  | 155,264 |  | 3 |
| Rocky River | 47.226 | -53.565 | 31 | 1990 | 2020 | 1,229 | 1,016 |  |  | 135 |  | 1,089 |  |  |  | 5 | 4 |
| Northeast River | 47.270 | -53.838 | 28 | 1974 | 2019 | 1,141 | 953 |  | 810 | 11 | 135 | 150 | 1 | 32 | 1 | 1 | 4 |
| Conne River | 47.868 | -55.706 | 36 | 1980 | 2020 | 8,072 | 6,640 |  | 2,208 | 3,903 |  |  | 4 | 16 | 1,941 |  | 4 |
| Harry's River | 48.507 | -58.507 | 27 | 1975 | 2015 | 1,932 | 1,142 |  | 61 | 1,826 |  |  |  | 45 |  |  | 4 |
| Terra Nova River | 48.668 | -54.003 | 34 | 1978 | 2021 | 5,916 | 3,520 |  | 1,408 | 86 |  | 4,265 |  | 39 |  | 118 | 4 |
| Rivière Saint-Jean | 48.774 | -64.425 | 34 | 1988 | 2021 | 10,343 | 4,297 | 4,203 | 9,749 |  |  |  | 78 | 288 |  |  | 5 |
| Middle Brook | 48.795 | -54.292 | 38 | 1978 | 2021 | 1,742 | 1,497 |  | 1,386 | 44 |  | 232 |  | 37 |  | 43 | 4 |
| Exploits River | 49.089 | -55.355 | 31 | 1983 | 2020 | 5,582 | 4,836 |  | 2,472 |  |  | 3,086 |  | 24 |  |  | 4 |
| Gander River | 49.260 | -54.499 | 37 | 1978 | 2021 | 4,062 | 3,656 |  | 3,758 | 54 |  | 214 |  | 36 |  |  | 4 |
| Rivière Trinité | 49.418 | -67.311 | 42 | 1980 | 2021 | 11,457 | 6,141 | 2,350 | 10,310 |  |  | 1,119 |  | 28 |  |  | 5 |
| Rivière aux Rochers | 50.023 | -66.874 | 23 | 1979 | 2004 | 5,722 | 2,180 | 2,409 | 1,389 |  |  | 3,421 |  |  |  | 365 | 6 |
| Torrent River | 50.613 | -57.169 | 26 | 1979 | 2007 | 2,225 | 1,856 |  | 443 | 23 |  | 1,743 |  | 16 |  |  | 4 |
| Western Arm Brook | 51.191 | -56.753 | 51 | 1971 | 2021 | 5,033 | 4,456 |  | 508 | 4,525 |  |  |  |  |  |  | 4 |
| Sand Hill River | 53.581 | -56.351 | 26 | 1970 | 2019 | 6,805 | 4,996 | 812 | 810 | 5,985 |  |  |  | 2 | 1 | 7 | 4 |
| English River | 54.963 | -59.762 | 21 | 1999 | 2019 | 3,216 | 2,507 |  | 33 | 3,166 |  |  |  | 17 |  |  | 4 |

^1^ Fishery-dependent sampling method.

^2^ Fishery-independent sampling method.

^3^ Other sampling methods included: bycatch (Harry’s); individuals found dead (Northeast, Conne, Terra Nova, Saint-Jean, Middle Brook, Exploits, Gander, Trinité, Torrent, Sand Hill, and English); and telemetry project (Saint-Jean).

^4^ Authors, affiliations and citations for data sources: 1) Daniela Notte, Andrew Taylor, Fisheries and Oceans Canada Maritime region; 2) J. Derek Hogan, Sherise McWilliam, Fisheries and Oceans Canada Maritime region; 3) Gérald Chaput, Scott Douglas, Kari Underhill, Fisheries and Oceans Canada Gulf region (Hayward et al., 2014); 4) Martha J. Robertson, Fisheries and Oceans Canada Newfoundland region; 5) Julien April, Ministère de l’Environnement, de la Lutte contre les changements climatiques, de la Faune et des Parcs (Cauchon and April, 2021); 6) 5) Julien April, Ministère de l’Environnement, de la Lutte contre les changements climatiques, de la Faune et des Parcs.


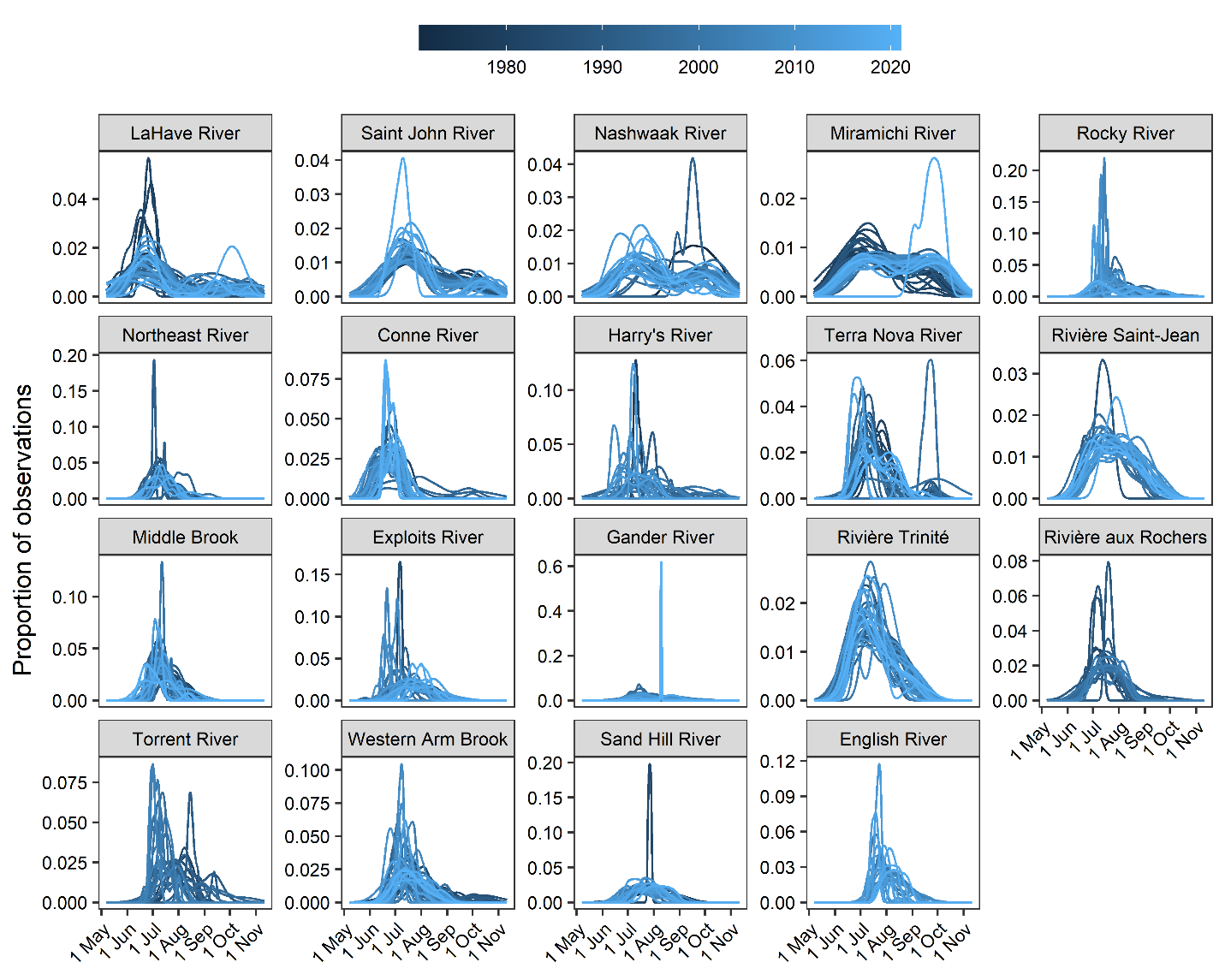


Figure S1. Density curves showing the sampling dates of Atlantic salmon (*Salmo salar*) with a known fork length for 19 rivers throughout Eastern Canada for each year from 1971-2021. The proportion of observations reflects the number of salmon within each river sampled on a given day in each year. Mean fork length/river was calculated for all years where a minimum of 10 1SW and/or 10 2SW salmon were sampled and these years were included in the Dynamic Factor Analysis (DFA).

## Variation in fork length


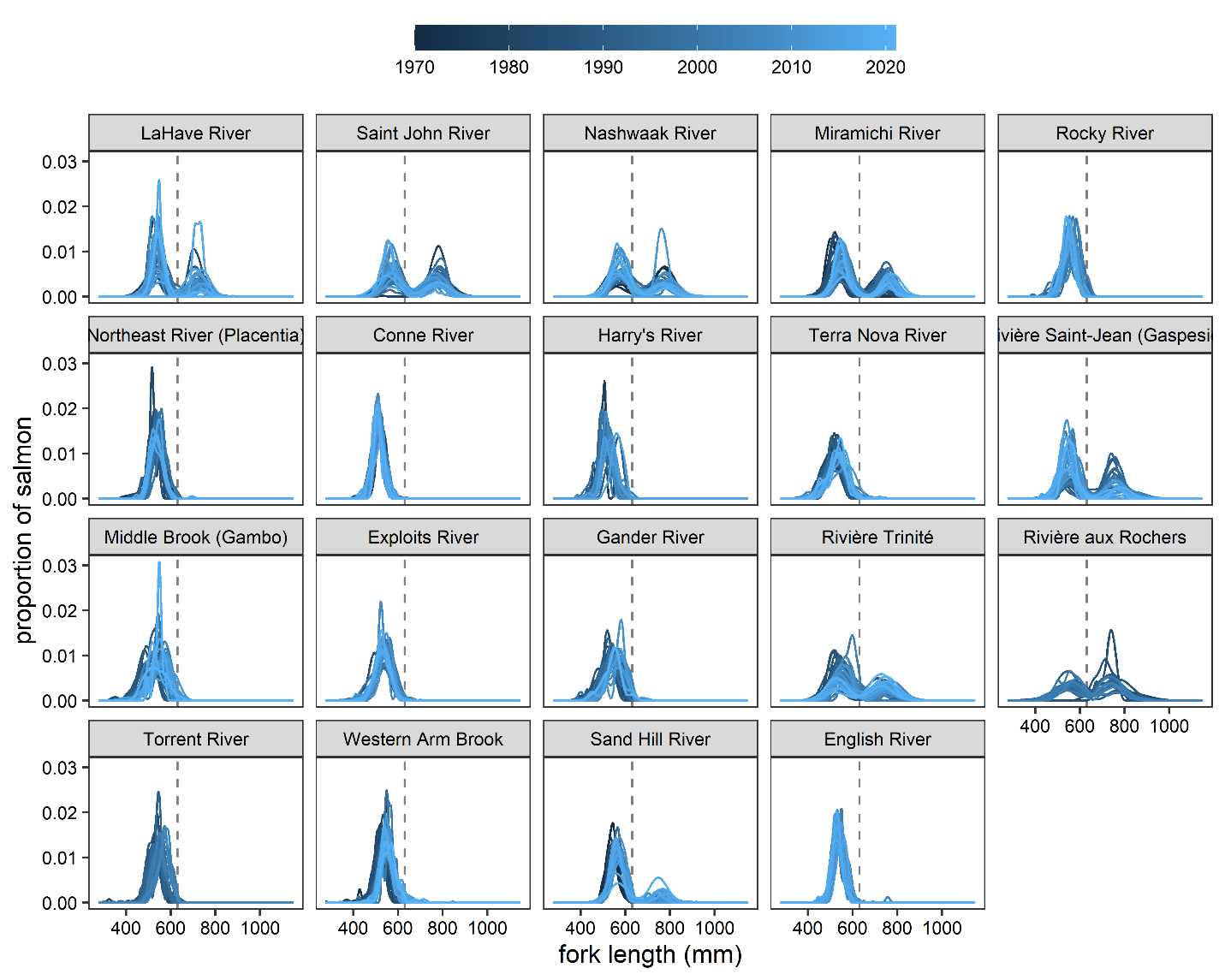


Figure S2. Density curves showing the annual distribution of fork length values for 1SW and 2SW Atlantic salmon (*Salmo salar*) for 19 rivers throughout Eastern Canada from 1971-2021. The vertical line at 630 mm is generally used as a threshold to separate most 1SW salmon from 2SW and older first-time and repeat spawners.


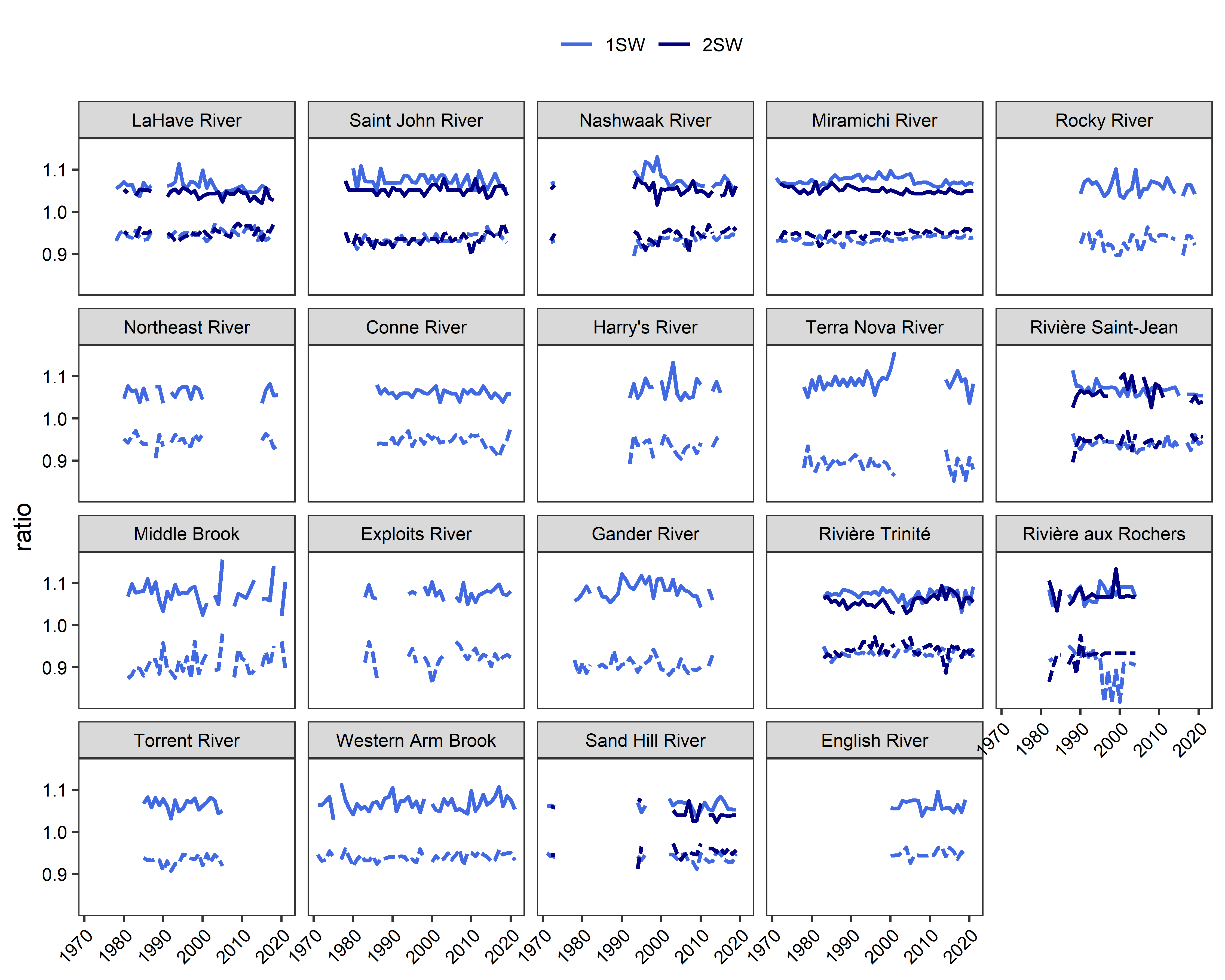


Figure S3. Ratio of the 10^th^ quartile to median fork length (dashed lines) and 90^th^ quartile to median fork length (solid lines) for 1SW and 2SW Atlantic salmon across 19 rivers throughout Eastern Canada from 1971-2021. Most rivers did not exhibit temporal patterns in these ratios indicating that the distribution of values was similar through time.


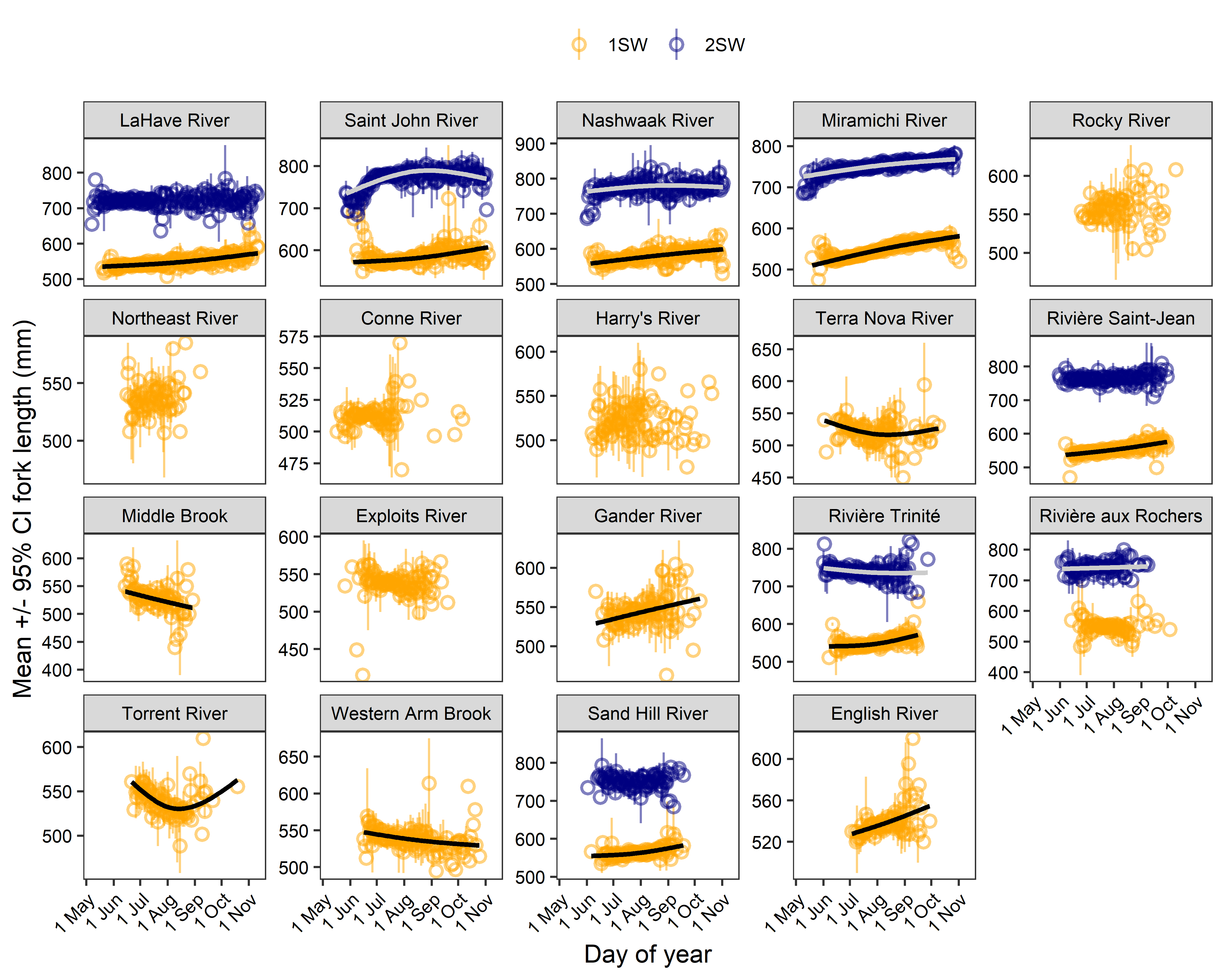


Figure S4. Relationships between mean annual fork length and day of year for Atlantic salmon (*Salmo salar*) from 19 rivers throughout Eastern Canada from 1971-2021. Trend lines were added for rivers where there was a significant relationship (P < 0.05) between mean annual fork length and day of year determined with a generalized additive model (GAM) with smooth terms for day of year by river for each age class.

## Covariate information

### Sources of covariate datasets

Table S2. Sources of data used as provided or used to calculate covariates that represent ocean conditions, thermal habitat, food availability, density-dependence at sea and fisheries harvest that may affect the fork length of Atlantic salmon (*Salmo salar*) in Eastern Canada.

| Covariate | Source |
| --- | --- |
| Atlantic Multidecadal Oscillation (AMO) | National Oceanic and Atmosphere Administration: <https://psl.noaa.gov/data/timeseries/AMO/> (accessed 31 October 2022) |
| North Atlantic Oscillation (NAO) | National Oceanic and Atmosphere Administration: <https://www.ncei.noaa.gov/access/monitoring/nao/> (accessed 31 October 2022) |
| Newfoundland and Labrador Climate Index (NLCI) | Cyr and Galbraith, 2021: <https://doi.org/10.20383/101.0301> |
| Hadley Centre Sea Ice and Sea Surface Temperature (HadISST1) dataset from the | UK’s Meteorological Office: <https://www.metoffice.gov.uk/hadobs/hadisst/> (accessed 2 December 2022) |
| Zooplankton | Johns, 2023 (Continuous Plankton Recorder (CPR) Survey, Marine Biological Association of the United Kingdom): <http://doi.org/10.17031/6513f104926b6> |
| Capelin | Aaron Adamack, Fisheries and Oceans Canada (unpubl. data): <http://doi.org/10.17605/OSF.IO/78PWT> |
| Pre-fishery abundance for North American Commission (NAC) and Southern Northeast Atlantic Commission (SNEAC) | International Council for the Exploration of the Sea (ICES 2023, Tables 3.3.4.4 and 4.3.6.1): [https://doi.org/10.17895/ices.pub.22743713.v2](https://can01.safelinks.protection.outlook.com/?url=https%3A%2F%2Fdoi.org%2F10.17895%2Fices.pub.22743713.v2&data=05%7C01%7CTara.Imlay%40dfo-mpo.gc.ca%7C4bd33782cec34f86b25808dbc0fc7029%7C1594fdaea1d94405915d011467234338%7C0%7C0%7C638315962664238030%7CUnknown%7CTWFpbGZsb3d8eyJWIjoiMC4wLjAwMDAiLCJQIjoiV2luMzIiLCJBTiI6Ik1haWwiLCJXVCI6Mn0%3D%7C3000%7C%7C%7C&sdata=tCnrY6qzLCACr7pwFhekISAubkmwZG070PB2HlyugvE%3D&reserved=0) |
| Atlantic salmon exploitation rates for fisheries at Newfoundland and Labrador, and Saint Pierre and Miquelon | Fisheries and Oceans Canada (unpubl. data): <http://doi.org/10.17605/OSF.IO/78PWT> |
| Atlantic salmon exploitation rates for fisheries at West Greenland | International Council for the Exploration of the Sea (ICES 2023, Figure 5.1.3.1 and Tables 5.2.2.5 and 4.3.6.1): [https://doi.org/10.17895/ices.pub.22743713.v2](https://can01.safelinks.protection.outlook.com/?url=https%3A%2F%2Fdoi.org%2F10.17895%2Fices.pub.22743713.v2&data=05%7C01%7CTara.Imlay%40dfo-mpo.gc.ca%7C4bd33782cec34f86b25808dbc0fc7029%7C1594fdaea1d94405915d011467234338%7C0%7C0%7C638315962664238030%7CUnknown%7CTWFpbGZsb3d8eyJWIjoiMC4wLjAwMDAiLCJQIjoiV2luMzIiLCJBTiI6Ik1haWwiLCJXVCI6Mn0%3D%7C3000%7C%7C%7C&sdata=tCnrY6qzLCACr7pwFhekISAubkmwZG070PB2HlyugvE%3D&reserved=0). |

### Indices of plankton and capelin availability used to determine food availability

We obtained zooplankton abundance data from the Continuous Plankton Recorder (CPR) Survey from 1958 to 2021 for our identified spatial regions for 1SW and 2SW salmon (Figure 3). The CPR is towed behind commercial vessels travelling between specific ports-of-call, and continuously samples plankton at a depth of about 7 m via water passing through a filtering silk (Richardson et al., 2006). The filtering silk from each route is divided into samples that correspond to 10 nautical miles of towing, and counts of different plankton taxa are conducted transection every second sample (Richardson et al., 2006). The counts are converted to semi-quantitative categorical abundance estimate (Richardson et al., 2006). To the extent possible, routes are sampled monthly; however, there were four years (1987-1990) when no surveys were conducted in either spatial regions. The CPR data was used to develop annual indices of zooplankton biomass (i.e., *Calanus* spp., including *Calanus* I-IV, *C. finmarchicus*, *C. glacialis*, *C. hyperboreus*, and *C. helgolandicus*; *Metridia* spp., including *Metridia* I-IV, *M. longa*, and *M. lucens*; *Oithona* spp.; Pseudocalanus, including *Pseudocalanus* spp. and *Para-Pseudocalanus spp*.; Order Euphausiacea, and Suborder Hyperiidea). We multiplied the abundance of each group by the mass (all taxa, except *Oithona* spp.: 0.08*length (mm)^2.1^, Richardson et al., 2006; *Oithona* spp.: 0.008*length (mm)^3^, Krylov, 1968). Lengths for copepods were reviewed in Richardson et al. (2006) and the length for *P. elongatus* was used for *Pseudocalanus* spp. taxon. A length for euphausiids in salmon diets was obtained from Figure 5 in Lindley (1978) and Williams and Robins (1981) found that 76% of one hyperiid amphipod (*Parathemisto gaudichaudi*) in CPR samples were < 3 mm in length (range: > 3 mm to <18 mm), therefore, we used 3 mm as the length for this group. Next, using the gam function in R package mcgv (Wood, 2017), we used a generalized additive model to determine the relationship between mean monthly zooplankton biomass and a smooth (bs = “tp”) for year, a cyclical smooth (bs = “cc) for month, and tensor product for mean latitude and longitude of sampling locations. Our final models in the spatial regions for first and second years explained 60.1% and 77.0%, respectively, of the deviance in monthly mean zooplankton biomass. Finally, we selected the most commonly sampled location (first year = 50.35°, -48.48°, second year = 55.70°, -39.79°; red symbols in Figure 3), predicted the monthly mean biomass at that location, and then summed the monthly values across each year (Figure S5A).

We obtained datasets on the estimated spring biomass of Capelin (*Mallotus villosus*) from two spring acoustic survey (A. Adamack, Fisheries and Oceans Canada) that cover an important nursery area for Capelin. The first survey covered the area around the Grand Banks, Newfoundland which included Northwest Atlantic Fisheries Organization (NAFO) divisions 3L, 3N, and 3O (3LNO). It was conducted in 17 years from 1975-1994, specifically in 1975-1979, 1981, 1983-1991, 1993, and 1994). The second survey covered NAFO division 3L, and since 1996, the southern part of 3K (3LK) that includes the waters from the northern and eastern parts of Newfoundland (Bourne et al., 2021; DFO, 2020). It was conducted in 29 years from 1982-2019, specifically 1982, 1985-1992, 1996, 1999-2005, 2007-2015, and 2017-2019 (Bourne et al., 2021; DFO, 2020). Both surveys are conducted during the spring, therefore we assumed that Capelin biomass was correlated with plankton indices in the previous year as this better represented the prey base. As there were gaps in each of the survey data for Capelin, we used the available data and our plankton indices in the 1SW polygon to impute the missing values for Capelin biomass in a PCA using the imputePCA function in the R package missMDA (Josse and Husson, 2016). The imputed values for the years with missing data were used in the PCA (Figure S5B).

Lastly, to determine the final index of food availability, we performed two PCAs using the zooplankton and Capelin biomass with imputed values for the years with missing data. For 1SW salmon, the index included zooplankton from the 1SW polygon and Capelin biomass in the subsequent year. For 2SW salmon, the same variables as the 1SW index during the first year at sea, as well as zooplankton from the 2SW in the second year and Capelin biomass in the subsequent year. The first principal component for 1SW and 2SW salmon accounted for 77.6% and 72.4% of the variability in these datasets, respectively. Lower values of this index represent periods when zooplankton and capelin biomass was lower (Figure S6).


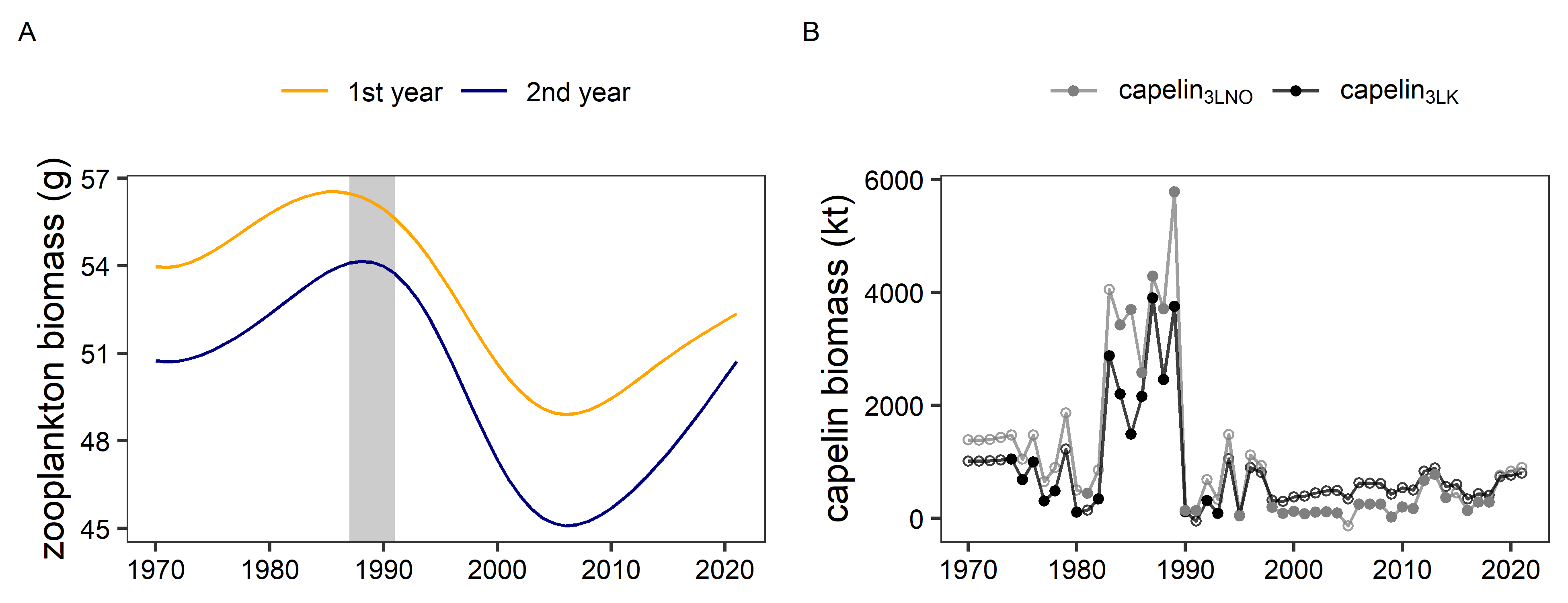
Figure S5. Biomass values for A) zooplankton using data from the Continuous Plankton Recorder (CPR) survey and B) Capelin (*Mallotus villosus*) biomass from two surveys in Northwest Atlantic Fisheries Organization (NAFO) divisions 3K, 3L, 3N, and 3O, and 3L and 3K around Newfoundland from 1970-2021. In A), the grey polygon indicates the four years (1987-1990) when no surveys were conducted, and in B) the open circles indicate imputed values and the closed circles indicate biomass estimates from acoustic the surveys.

Table S3. Loadings from Principal Components Analyses to determine food availability for 1SW and 2SW Atlantic salmon (*Salmo salar*) from Eastern Canada.

|  | Covariate^1^ | PC1 | PC2 | PC3 | PC4 | PC5 | PC6 |
| --- | --- | --- | --- | --- | --- | --- | --- |
| 1SW^2^ | Zooplankton_1_ | 0.69 | 0.71 | 0.15 |  |  |  |
|  | Capelin 3LNO_1_ | 0.42 | -0.56 | 0.72 |  |  |  |
|  | Capelin 3LK_1_ | 0.59 | -0.43 | -0.68 |  |  |  |
|  |  |  |  |  |  |  |  |
| 2SW^3^ | Zooplankton_1_ | 0.53 | 0.45 | 0.02 | 0.56 | 0.09 | 0.44 |
|  | Zooplankton_2_ | 0.26 | -0.43 | 0.47 | 0.37 | -0.57 | -0.25 |
|  | Capelin 3LNO_1_ | 0.39 | -0.37 | 0.5 | -0.37 | 0.51 | 0.26 |
|  | Capelin 3LK_1_ | 0.53 | 0.46 | 0.03 | -0.41 | -0.09 | -0.58 |
|  | Capelin 3LNO_2_ | 0.27 | -0.4 | -0.48 | 0.36 | 0.46 | -0.43 |
|  | Capelin 3LK_2_ | 0.39 | -0.31 | -0.55 | -0.34 | -0.43 | 0.39 |

^1^ Subscripts indicate whether the covariate is from the first or second year at sea.

^2^ Proportion of variance accounted for by PC1-PC3: 0.78, 0.22, and 0.01.

^3^ Proportion of variance accounted for by PC1-PC6: 0.72, 0.16, 0.10, 0.004, 0.004, and 0.003.


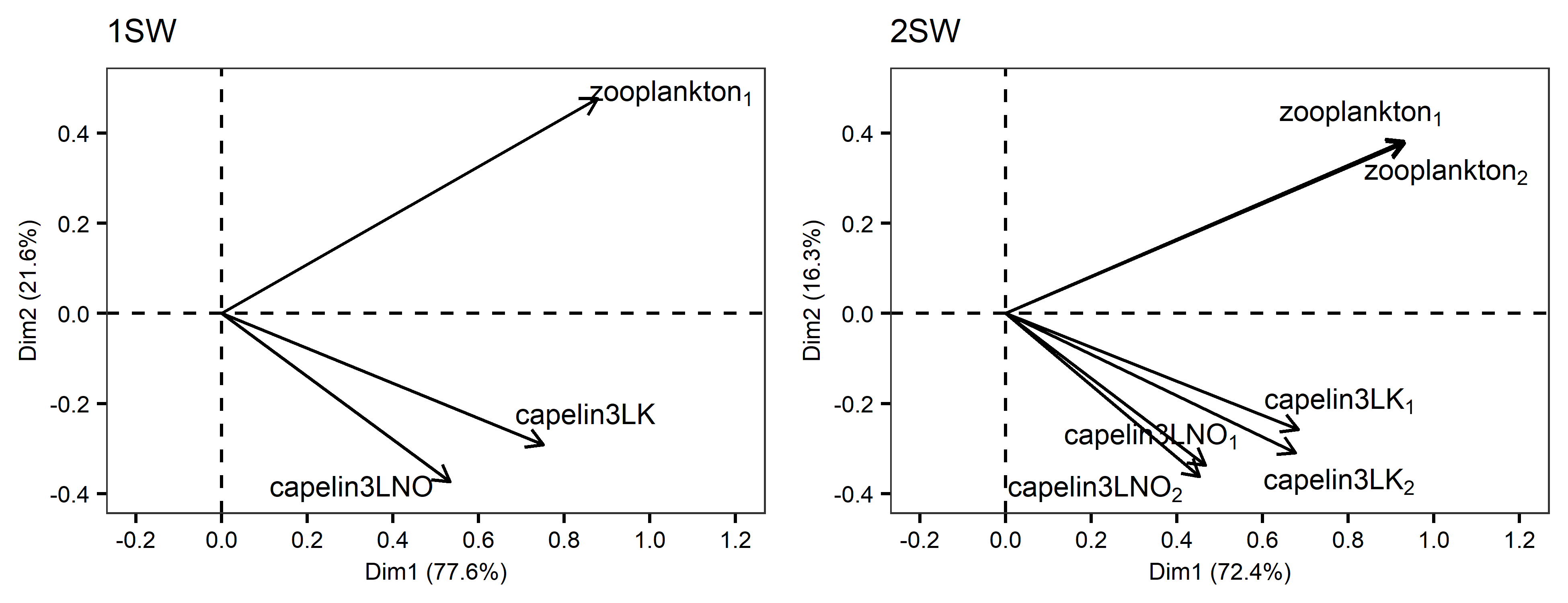


Figure S6. Plot of the principal components analysis (PCA) eigenvectors for annual indices of zooplankton and capelin biomass used to derive an overall index of food availability for 1SW and 2SW Atlantic salmon (*Salmo salar*) in Eastern Canada. Subscripts indicate whether the covariate is related to the first or second year at sea.

### Exploitation rates for NAC and West Greenland fisheries


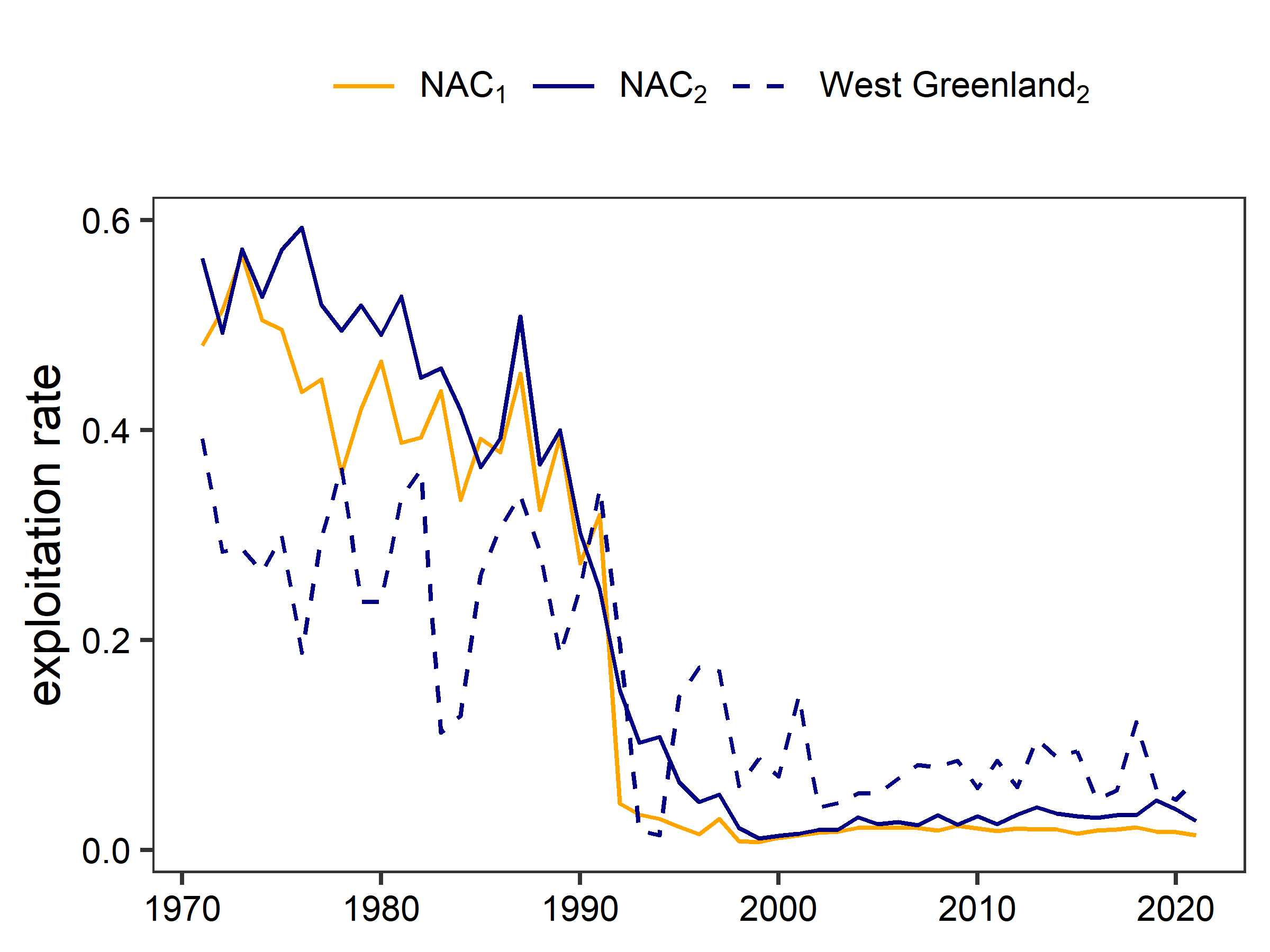


Figure S7. Exploitation rates for North American salmon harvested in the mixed-stock marine fisheries around Newfoundland and Labrador, and Saint Pierre and Miquelon (collectively referred to as North American Commission or NAC fisheries) and West Greenland. Colors and subscripts indicate whether the covariate is related to the first or second year at sea.

### Variance Inflation Factors (VIFs) and pair-wise correlations between covariates

Prior to including these covariates, we first identified whether there were variables with Variance Inflation Factors (VIF > 5) (Zuur et al., 2009) or strong Pearson correlations (Pearson’s r > |0.7|) between pairs of covariates. For 1SW salmon, three covariates (winter thermal habitat, pre-fishery abundance and exploitation rate) had VIF > 5 (Table S4). AMO was strongly correlated with winter thermal habitat (r = 0.77) and exploitation rate (r = -0.75) (Figure S8). Winter thermal habitat, food availability, and pre-fishery abundance were all strongly correlated with exploitation rate (r = -0.71, 0.73 and 0.88, respectively). For 2SW salmon, six covariates had VIFs > 5, including AMO during the second year, NLCI during the first year, winter thermal during the first year, pre-fishery abundance during the first and second year, and exploitation rate for the NAC fisheries (Table S5). AMO in the first and second years were strongly correlated (r = 0.74), as well as winter thermal habitat in the first year (r = 0.73 and r = 0.76, respectively), and exploitation rate for the NAC fisheries (r = -0.78 and -0.76) (Figure S9). AMO in the first year was also strongly correlated with pre-fishery abundance in the second year (r = -0.73) and AMO in the second year was strongly correlated with food availability (r = -0.71). NLCI during the first year was strongly correlated with winter thermal habitat in the first year (r = 0.73). Winter thermal habitat in the first year was strongly correlated with food availability (r = -0.71). Food availability was strongly correlated with exploitation rate for the West Greenland fishery (r = 0.72). Pre-fishery abundance in the first and second years were strongly correlated (r = 0.81); they were also correlated with the exploitation rate for NAC (r = 0.91 and 0.90) and West Greenland (r = 0.76 and 0.71) fisheries in the second year. Lastly, exploitation rates were strongly correlated between the NAC and West Greenland fisheries (r = 0.82).

Table S4. Variance inflation factors (VIFs) for covariates that may be related to changes in the body size of 1SW Atlantic salmon (*Salmo salar*) from Eastern Canada. VIFs > 5 indicate covariates that are correlated with one or more other covariates.

| Group | Covariate | VIF |
| --- | --- | --- |
| Climate | Atlantic Multidecadal Oscillation (AMO) | 3.57 |
|  | North Atlantic Oscillation (NAO) | 2.08 |
|  | Newfoundland and Labrador Climate Index (NLCI) | 2.21 |
| Thermal habitat | Summer thermal habitat | 2.77 |
|  | Winter thermal habitat | 8.30 |
| Food availability | Food availability | 2.85 |
| Density-dependence | Pre-fishery abundance (PFA) | 5.43 |
| Harvest | Exploitation rate of NAC fisheries | 12.44 |

Table S5. Variance inflation factors (VIFs) for covariates that may be related to changes in the body size of 2SW Atlantic salmon (*Salmo salar*) from Eastern Canada. VIFs > 5 indicate covariates that are correlated with one or more other covariates. Subscripts indicate whether the covariate is related to the first or second year at sea.

| Group | Covariate | VIF |
| --- | --- | --- |
| Climate | Atlantic Multidecadal Oscillation (AMO)_1_ | 4.55 |
|  | Atlantic Multidecadal Oscillation (AMO)_2_ | 5.20 |
|  | North Atlantic Oscillation (NAO)_1_ | 3.19 |
|  | North Atlantic Oscillation (NAO)_2_ | 2.58 |
|  | Newfoundland and Labrador Climate Index (NLCI)_1_ | 5.44 |
|  | Newfoundland and Labrador Climate Index (NLCI)_2_ | 3.32 |
| Thermal habitat | Summer thermal habitat_1_ | 3.50 |
|  | Winter thermal habitat_1_ | 12.73 |
|  | Summer thermal habitat_2_ | 3.00 |
|  | Winter thermal habitat_2_ | 3.71 |
| Food availability | Food availability | 4.75 |
| Density-dependence | Pre-fishery abundance (PFA)_1_ | 8.59 |
|  | Pre-fishery abundance (PFA)_2_ | 8.82 |
| Harvest | Exploitation rate of NAC fisheries_2_ | 30.06 |
|  | Exploitation rate of West Greenland fishery_2_ | 4.01 |


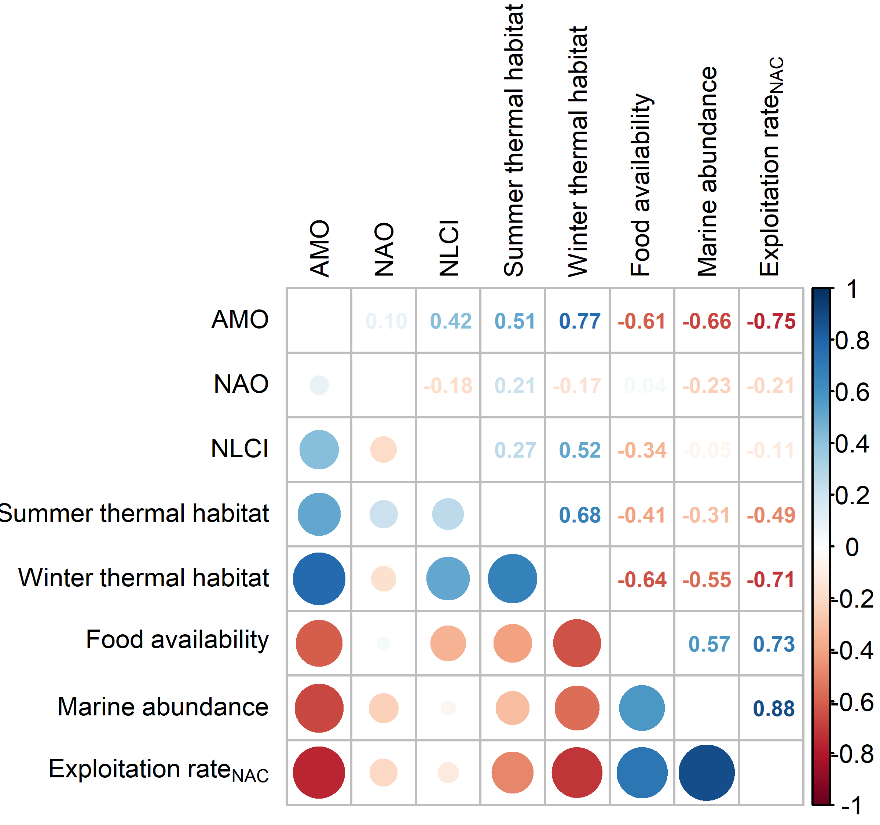


Figure S8. Pearson r correlation plot for eight covariates considered for their potential effect on the fork length of 1SW Atlantic salmon (*Salmo salar*) from Eastern Canada. Abbreviations: AMO = Atlantic Multidecadal Oscillation; NAO = North Atlantic Oscillation; and NLCI = Newfoundland and Labrador Climate Index.


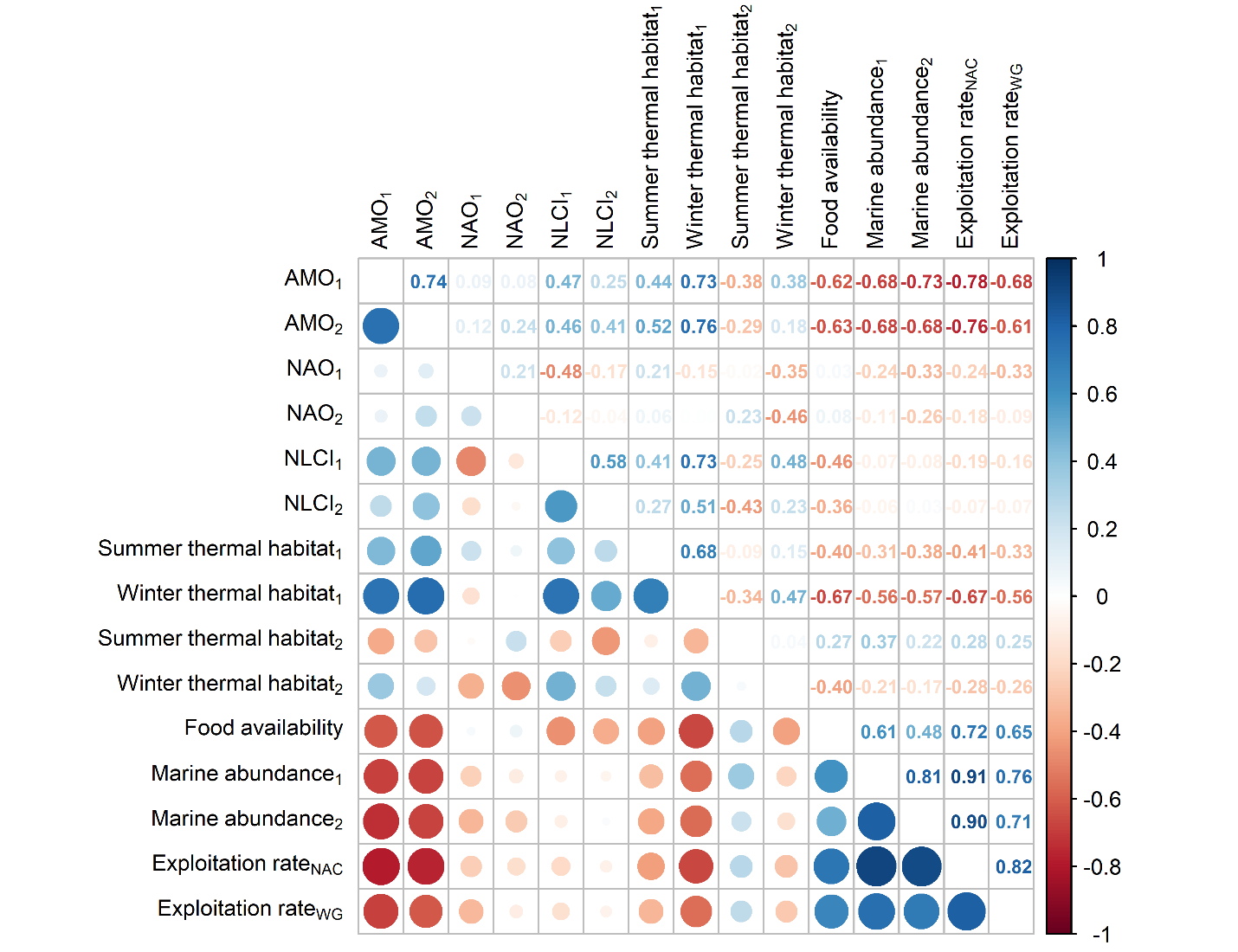


Figure S9. Pearson r correlation plot for 15 covariates considered for their potential effect on the fork length of 1SW Atlantic salmon (*Salmo salar*) from Eastern Canada. Abbreviations: AMO = Atlantic Multidecadal Oscillation; NAO = North Atlantic Oscillation; and NLCI = Newfoundland and Labrador Climate Index. The numbers after the name of each covariate indicate whether it was from the first or second year at sea.

## Dynamic factor analysis (DFA) information

### DFA model selection for 1SW and 2SW models without covariates

Table S6. Model comparison based on AICc for 12 dynamic factor models without covariates for 1SW and 2SW Atlantic salmon (*Salmo salar*) from distinct rivers and runs in Eastern Canada. The top model for 1SW and 2SW salmon is bolded, and 2SW models without a ΔAIC*c* or AIC*c* weight did not converge.

|  | # of common trends | Variance-covariance structure | ΔAIC*c*^1^ | AIC*c* weight | Log Likelihood |
| --- | --- | --- | --- | --- | --- |
| 1SW | **3** | **equalvarcov** | **0** | **0.91** | **-2260.93** |
|  | 4 | equalvarcov | 4.73 | 0.09 | -2243.37 |
|  | 2 | equalvarcov | 26.25 | <0.01 | -2294.03 |
|  | 4 | diagonal and equal | 33.79 | <0.01 | -2259.17 |
|  | 3 | diagonal and unequal | 36.05 | <0.01 | -2257.74 |
|  | 4 | diagonal and unequal | 36.53 | <0.01 | -2236.77 |
|  | 3 | diagonal and equal | 43.37 | <0.01 | -2283.83 |
|  | 2 | diagonal and unequal | 56.44 | <0.01 | -2289.15 |
|  | 2 | diagonal and equal | 66.57 | <0.01 | -2315.33 |
|  | 1 | diagonal and unequal | 126.25 | <0.01 | -2345.17 |
|  | 1 | equalvarcov | 139.39 | <0.01 | -2370.51 |
|  | 1 | diagonal and equal | 163.96 | <0.01 | -2383.87 |
|  |  |  |  |  |  |
| 2SW | **1** | **diagonal and unequal** | **0** | **0.51** | **-1036.83** |
|  | 3 | equalvarcov | 1.89 | 0.20 | -1029.59 |
|  | 3 | diagonal and unequal | 2.14 | 0.17 | -1022.32 |
|  | 3 | diagonal and equal | 4.50 | 0.05 | -1032.09 |
|  | 2 | diagonal and unequal | 4.69 | 0.05 | -1030.99 |
|  | 2 | equalvarcov | 8.07 | 0.01 | -1039.73 |
|  | 1 | equalvarcov | 10.07 | <0.01 | -1048.53 |
|  | 4 | diagonal and unequal | 11.68 | <0.01 | -1020.63 |
|  | 1 | diagonal and equal | 15.67 | <0.01 | -1052.40 |
|  | 2 | diagonal and equal | 23.35 | <0.01 | -1048.51 |
|  | 4 | diagonal and equal | - | - | - |
|  | 4 | equalvarcov | - | - | - |

^1^ AIC*c* values for the 1SW and 2SW models were 4,809.14 and 2123.51, respectively.

### Loadings for 1SW and 2SW base DFA models

Table S7. Loadings for the common trends from the dynamic factor analyses that examined changes in 1SW and 2SW Atlantic salmon (*Salmo salar*) fork length from 1971 or 1972 to 2021 throughout Eastern Canada. Rivers are arranged in order of increasing latitude and for the 1SW DFA, the common trend with the strongest loading (i.e., highest absolute value) is bolded.

|  | Loadings for common trends | | | |
| --- | --- | --- | --- | --- |
|  | 1SW | | | 2SW |
| River | 1 | 2 | 3 | 1 |
| LaHave River | **2.30** | -0.90 | -0.42 | 1.11 |
| Saint John River | **2.34** | 1.93 | -0.34 | -0.35 |
| Nashwaak River | **3.24** | 0.22 | -0.54 | 1.53 |
| Miramichi River | **3.78** | 0.32 | 3.20 | 5.54 |
| Rocky River | -0.05 | **1.78** | 0.29 |  |
| Northeast River | 1.07 | 1.00 | **1.76** |  |
| Conne River | -0.17 | **1.85** | 0.72 |  |
| Harry's River | -1.57 | **-4.44** | 0.91 |  |
| Terra Nova River | 0.14 | -0.34 | **2.86** |  |
| Rivière Saint-Jean | -1.63 | **4.89** | 2.66 | 5.88 |
| Middle Brook | -2.24 | 1.53 | **5.61** |  |
| Exploits River | **1.71** | 1.21 | 0.48 |  |
| Gander River | -1.74 | 1.32 | **4.81** |  |
| Rivière Trinité | 3.85 | **6.70** | 3.13 | 5.60 |
| Rivière aux Rochers | -0.66 | **14.61** | -3.59 | 0.77 |
| Torrent River | 0.27 | 2.60 | **3.66** |  |
| Western Arm Brook | 1.12 | -0.60 | **3.08** |  |
| Sand Hill River | **1.66** | -1.02 | 0.94 | 1.21 |
| English River | -0.83 | **1.25** | 0.46 |  |

### DFA model selection for 1SW and 2SW models with covariates


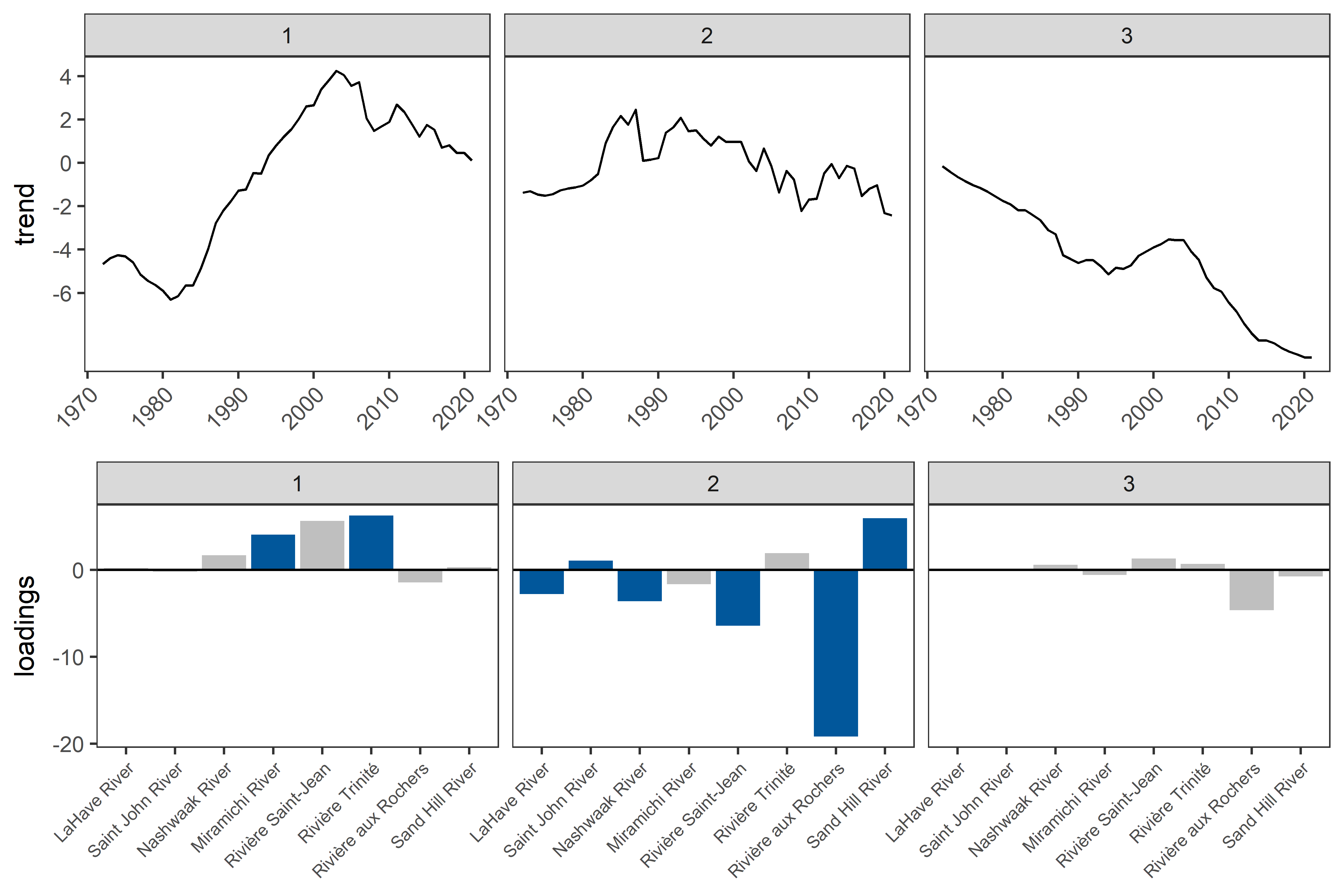


Figure S10. The three common trends and loadings for the dynamic factor analysis with the second lowest AIC that examined changes in 2SW Atlantic salmon fork length from 1971-2021 throughout Eastern Canada without accounting for covariates (base model). Rivers are arranged in order of increasing latitude. Trends and loadings are dimension-less, and blue bars represent the highest loading across trends for each river.

### DFA model selection for 1SW and 2SW models with covariates

Table S8. Model selection table for the relationships between climate indices, thermal habitat area, food availability, density dependence, and fisheries harvest and the body size of Atlantic salmon in Eastern Canada from 1971-2021 (1SW) or 1972-2021 (2SW).

|  | Model_1,2_ | ΔAIC*c*_3_ | Log Likelihood |
| --- | --- | --- | --- |
| 1SW | Exploitation rate (NAC) | -9.74 | -2232.26 |
|  | Base (no covariates) | 0 | -2260.93 |
|  | Pre-fishery abundance | 1.96 | -2238.11 |
|  | AMO | 2.99 | -2238.63 |
|  | Winter thermal habitat | 7.15 | -2240.71 |
|  | NLCI | 15.51 | -2244.89 |
|  | Summer thermal habitat | 21.73 | -2248.00 |
|  | Food availability | 29.17 | -2251.72 |
|  | NAO | 33.99 | -2254.13 |
|  |  |  |  |
| 2SW | Winter thermal habitat_2_ | -12.27 | -1021.30 |
|  | Exploitation rate (NAC) | -5.06 | -1024.91 |
|  | AMO_1_ | -5.03 | -1024.93 |
|  | NLCI_1_ | -4.31 | -1025.29 |
|  | NLCI_2_ | -3.51 | -1025.69 |
|  | NAO_1_ | -1.53 | -1026.68 |
|  | Base (no covariates) | 0 | -1036.83 |
|  | Pre-fishery abundance_2_ | 0.34 | -1027.61 |
|  | AMO_2_ | 1.08 | -1027.98 |
|  | Exploitation rate (WG) | 1.10 | -1027.99 |
|  | Pre-fishery abundance_1_ | 1.22 | -1028.05 |
|  | Food availability | 2.43 | -1028.65 |
|  | Summer thermal habitat_1_ | 3.01 | -1028.95 |
|  | NAO_2_ | 4.10 | -1029.49 |
|  | Winter thermal habitat_1_ | 4.48 | -1029.68 |
|  | Summer thermal habitat_2_ | 6.72 | -1030.80 |

### Fits for 1SW and 2SW DFA models with covariates


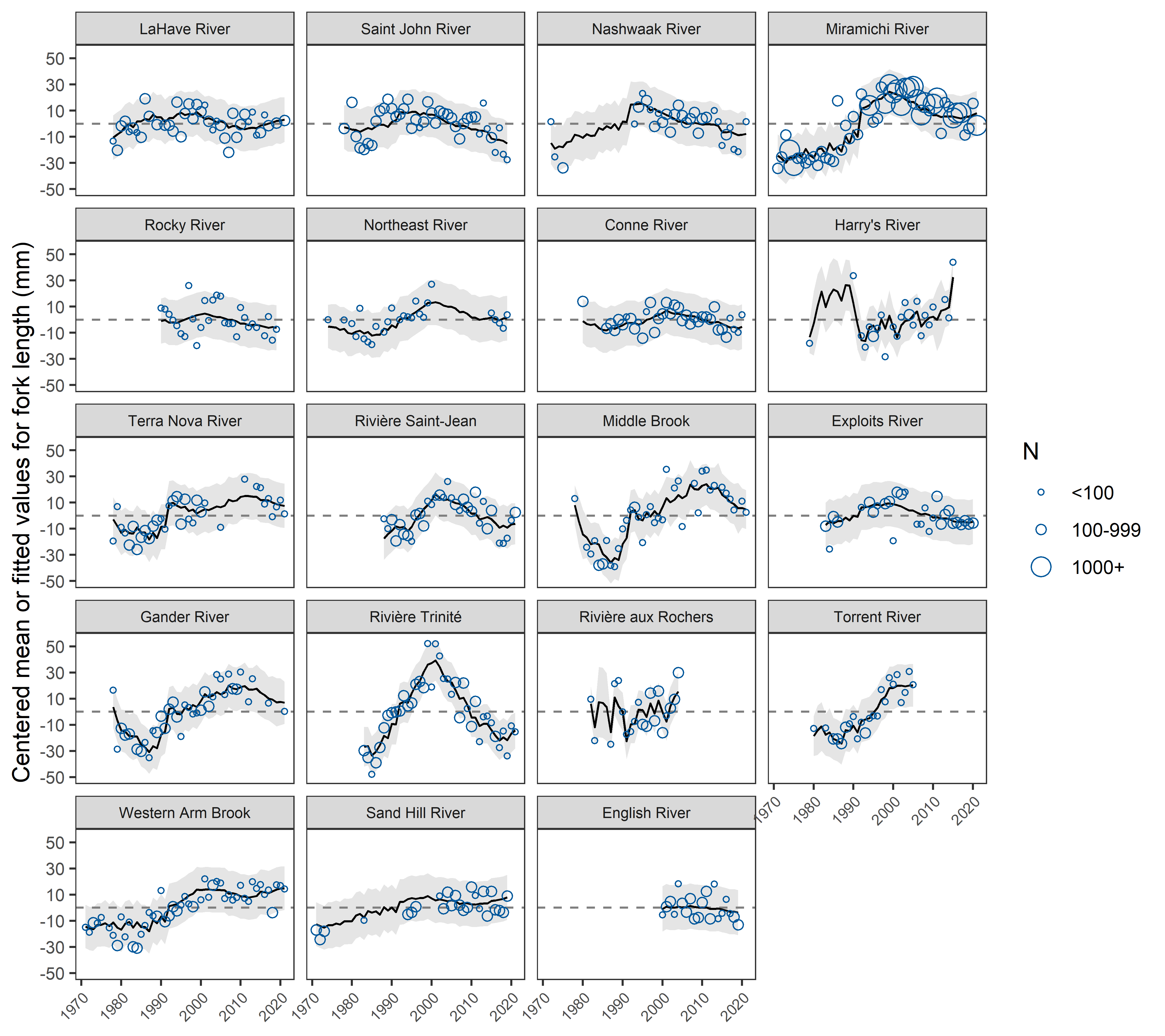


Figure S11. Predicted values ± 95% CIs for 1SW Atlantic salmon (*Salmo salar*) fork length from 1971-2020 for rivers across Eastern Canada after incorporating the exploitation rate for small salmon in the Newfoundland and Labrador, and St. Pierre and Miquelon (NAC) fisheries. The blue points represent the mean fork length for each timeseries centered around the river-specific mean when data was available and the size of these points reflect the sample size used to calculate the mean (N). Rivers are arranged in order of increasing latitude.


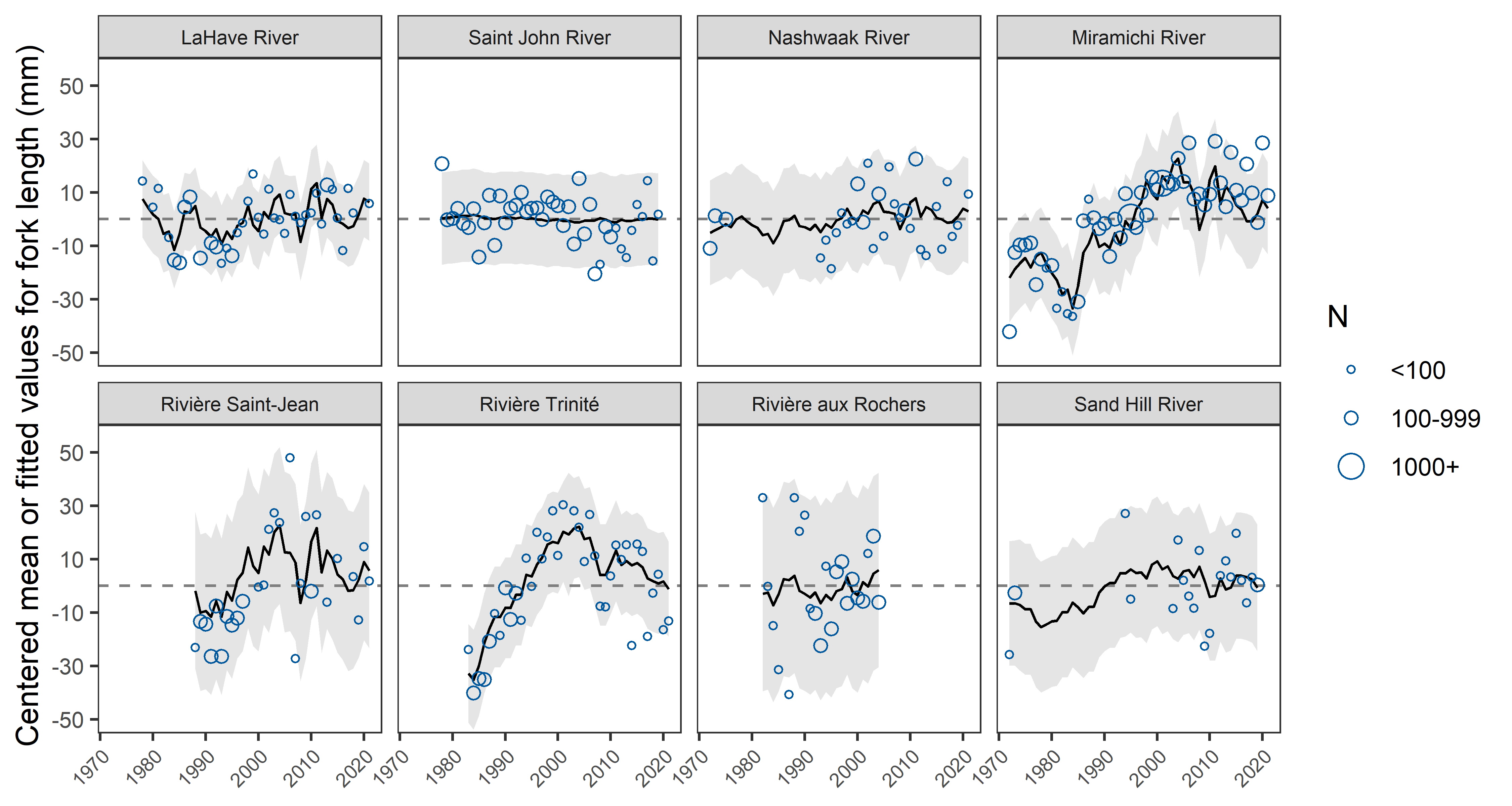


Figure S12. Predicted values ± 95% CIs for 2SW Atlantic salmon (*Salmo salar*) fork length from 1972-2021 for rivers across Eastern Canada after incorporating winter thermal habitat in the second year. The blue points represent the mean fork length for each timeseries centered around the river-specific mean when data was available and the size of these points reflect the sample size used to calculate the mean (N). Rivers are arranged in order of increasing latitude.

## Generalized additive model (GAM) information

The base DFA models for 1SW and 2SW salmon indicated periods of increasing fork length during the 1980s and 1990s (see Results). Additionally, in the 1SW DFA, two of the three common trends indicated that fork length declined in the early 2000s. Therefore, we created three subsets of the mean fork length values (centered) used in the DFA: mean fork length of 1SW salmon from 1) 1980-1999 and 2) 2000-2021, and 3) mean fork length of 2SW salmon from 1980-1999. Within each subset, we only included rivers with at least 10 years of data. Then, we modeled fork length during each period and for each age group with a GAM that included a smooth term for the year by river, i.e., s(year, by = river), with the R package mgcv (Wood, 2017). As we were focused on the overarching trend during these periods, we restricted the 'wiggliness’ of these smooths with the basis dimension (k = 4). For each GAM, we examined QQ-plots and plots of residuals vs. fitted values to ensure the final model was a good fit. We identified all rivers with a significant smooth (p < 0.05) (Table S9) and show the trends for these rivers during each period (Figures S13 and S14).

Table S9. Trends in mean fork length for 1SW and 2SW Atlantic salmon (*Salmo salar*) returns for rivers throughout Eastern Canada in two time periods (1980-1999 and 2000-2021). Rivers with significant p-values, i.e., p < 0.05, are bolded. Rivers are arranged in order of increasing latitude.

| Age | Period | River | P-value |
| --- | --- | --- | --- |
| 1SW | 1980-1999 | LaHave River | 0.27 |
|  |  | Saint John River | 0.13 |
|  |  | **Miramichi River** | **<0.001** |
|  |  | Rocky River | 0.67 |
|  |  | **Northeast River** | **0.02** |
|  |  | Conne River | 0.89 |
|  |  | **Terra Nova River** | **<0.001** |
|  |  | Rivière Saint-Jean | 0.22 |
|  |  | **Middle Brook** | **<0.001** |
|  |  | **Exploits River** | **0.01** |
|  |  | **Gander River** | **<0.001** |
|  |  | **Rivière Trinité** | **<0.001** |
|  |  | Rivière aux Rochers | 0.37 |
|  |  | **Torrent River** | **<0.001** |
|  |  | **Western Arm Brook** | **<0.001** |
|  |  |  |  |
| 1SW | 2000-2021 | LaHave River | 0.20 |
|  |  | Saint John River | 0.50 |
|  |  | Nashwaak River | 0.87 |
|  |  | Miramichi River | 0.19 |
|  |  | Rocky River | 0.27 |
|  |  | Conne River | 0.32 |
|  |  | Harry's River | 0.51 |
|  |  | Rivière Saint-Jean | 0.36 |
|  |  | Gander River | 0.06 |
|  |  | **Rivière Trinité** | **<0.001** |
|  |  | Western Arm Brook | 0.76 |
|  |  | English River | 0.84 |
|  |  |  |  |
| 2SW | 1980-1999 | LaHave River | 0.25 |
|  |  | Saint John River | 0.59 |
|  |  | **Miramichi River** | **<0.001** |
|  |  | Rivière Saint-Jean | 0.05 |
|  |  | **Rivière Trinité** | **<0.001** |
|  |  | Rivière aux Rochers | 0.92 |


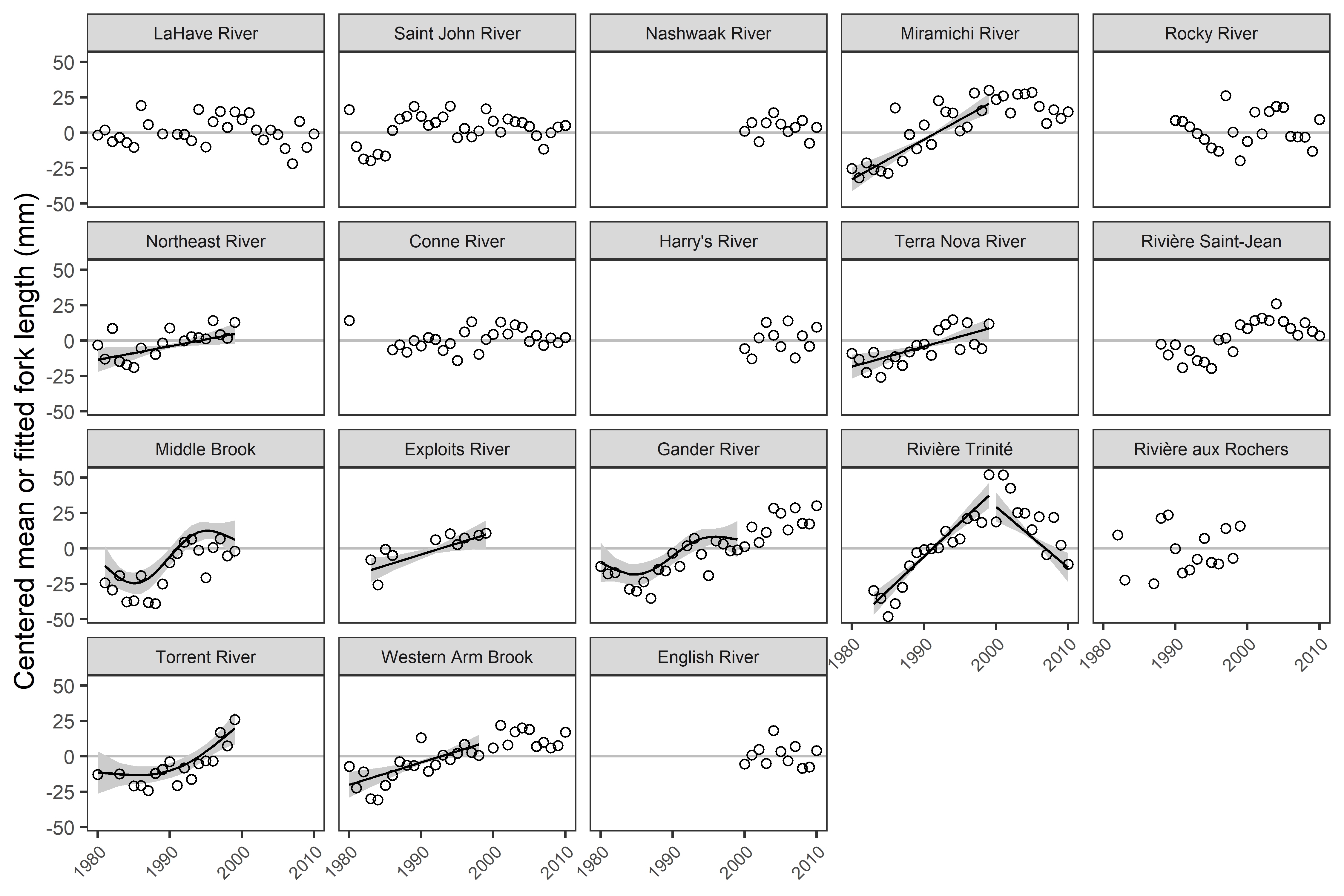


Figure S13. Predicted values ± 95% CIs for 1SW Atlantic salmon (*Salmo salar*) fork length for rivers across Eastern Canada using generalized additive models (GAM) for data from 1980-1999 and 2000-2021. GAM results are shown if the relationship was significant for that river (p < 0.05, Table S9). The black points represent the mean fork length for each timeseries centered around the river-specific mean. Rivers are arranged in order of increasing latitude.


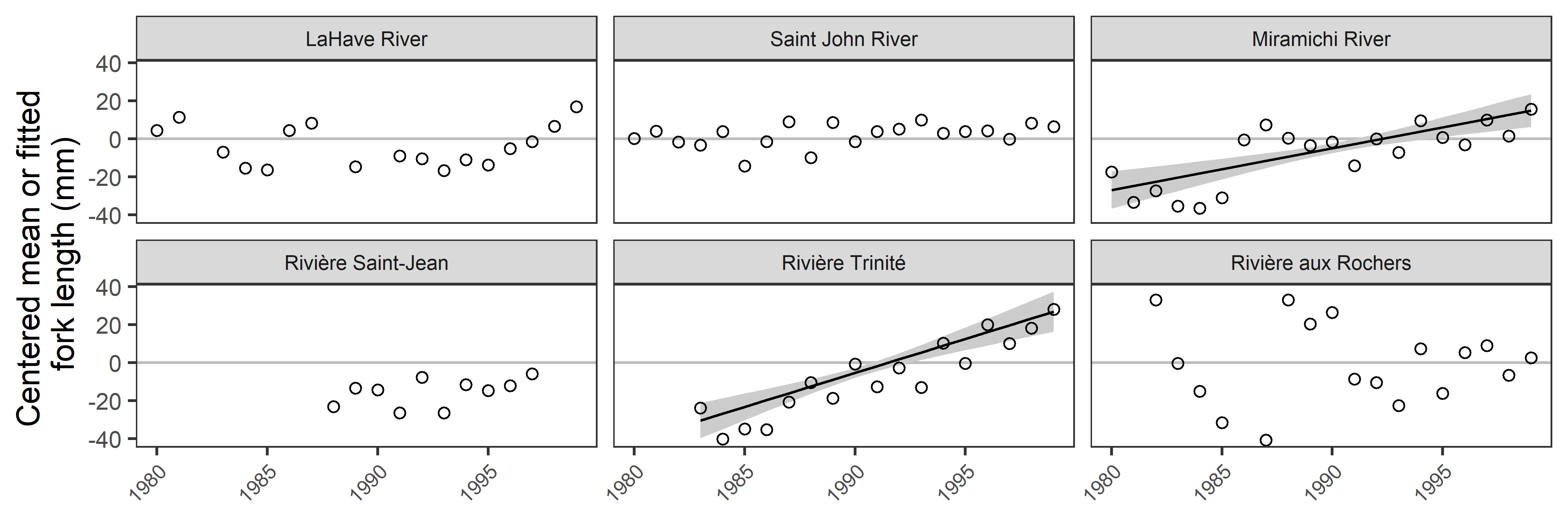


Figure S14. Predicted values ± 95% CIs for 2SW Atlantic salmon (*Salmo salar*) fork length for rivers across Eastern Canada from 1980-1999 using a generalized additive model (GAM). GAM results are shown if the relationship was significant for that river (p < 0.05, Table S9). The black points represent the mean fork length for each timeseries centered around the river-specific mean. Rivers are arranged in order of increasing latitude.

## References

Bourne, C., Murphy, H., Adamack, A.T., Lewis, K., 2021. Assessment of Capelin (*Mallotus villosus*) in 2J3KL to 2018 (No. 2021/055), Canadian Science Advisory Secretariat Research Document. Fisheries and Oceans Canada, St. John’s, NL.

Cauchon, V., April, J., 2021. Suivi des populations témoins de saumon atlantique au Québec: Rapport scientifique 2021. Ministère des Forêts, de la Faune et des Parcs, Secteur de la faune et des parcs.

Cyr, F., Galbraith, P.S., 2021. A climate index for the Newfoundland and Labrador shelf. Earth Syst. Sci. Data 13, 1807–1828. https://doi.org/10.5194/essd-13-1807-2021

DFO, 2020. Assessment of 2J3KL Capelin in 2020 (No. 2022/013), Canadian Science Advisory Secretariat Science Advisory Report. Fisheries and Oceans Canada.

Hayward, J., Sheasgreen, J., Douglas, S., Reid, J., 2014. Diadromous fish monitoring programs in the Miramichi River system in 2011 (No. 3042), Canadian Manuscript Report of Fisheries and Aquatic Sciences.

ICES, 2023. Working Group on North Atlantic Salmon (WGNAS). ICES Sci. Rep. 5, 478 pp. https://doi.org/10.17895/ices.pub.22743713

Johns, D., 2023. Selected NW Atlantic CPR taxa. The Archive for Marine Species and Habitats Data (DASSH). (Dataset). https://doi.org/10.17031/6513f104926b6

Josse, J., Husson, F., 2016. missMDA: A Package for Handling Missing Values in Multivariate Data Analysis. J. Stat. Softw. 70, 1–30. https://doi.org/10.18637/jss.v070.i01

Krylov, V.V., 1968. Relation between wet formalin weight of copepods and copepod body length. Oceanology 8, 723–727.

Lindley, J.A., 1978. Population Dynamics and Production of Euphausiids. I. *Thysanoessa longicaudata* in the North Atlantic Ocean. Mar. Biol. 46, 121–130.

Richardson, A.J., Walne, A.W., John, A.W.G., Jonas, T.D., Lindley, J.A., Sims, D.W., Stevens, D., Witt, M., 2006. Using continuous plankton recorder data. Prog. Oceanogr. 68, 27–74. https://doi.org/10.1016/j.pocean.2005.09.011

Williams, R., Robins, D., 1981. Seasonal Variability in Abundance and Vertical Distribution of *Parathemisto gaudichaudi* (Amphipoda: Hyperiidea) in the North East Atlantic Ocean. Mar. Ecol. Prog. Ser. 4, 289–298. https://doi.org/10.3354/meps004289

Wood, S.N., 2017. Generalized Additive Models: An Introduction with R (2nd edition). Chapman and Hall.

Zuur, A.F., Ieno, E.N., Walker, N.J., Saveliev, A.A., Smith, G.M., 2009. Mixed effects models and extensions in ecology with R. Springer, New York.
